# Novel Structural Components of the Ventral Disc and Lateral Crest in *Giardia intestinalis*

**DOI:** 10.1371/journal.pntd.0001442

**Published:** 2011-12-20

**Authors:** Kari D. Hagen, Matthew P. Hirakawa, Susan A. House, Cindi L. Schwartz, Jonathan K. Pham, Michael J. Cipriano, Moises J. De La Torre, Albert C. Sek, Gary Du, Brystal M. Forsythe, Scott C. Dawson

**Affiliations:** 1 Department of Microbiology, University of California Davis, Davis, California, United States of America; 2 Boulder Laboratory for 3D Electron Microscopy of Cells, Department of Molecular, Cellular and Developmental Biology, University of Colorado, Boulder, Colorado, United States of America; University of Queensland, Australia

## Abstract

*Giardia intestinalis* is a ubiquitous parasitic protist that is the causative agent of giardiasis, one of the most common protozoan diarrheal diseases in the world. *Giardia* trophozoites attach to the intestinal epithelium using a specialized and elaborate microtubule structure, the ventral disc. Surrounding the ventral disc is a less characterized putatively contractile structure, the lateral crest, which forms a continuous perimeter seal with the substrate. A better understanding of ventral disc and lateral crest structure, conformational dynamics, and biogenesis is critical for understanding the mechanism of giardial attachment to the host. To determine the components comprising the ventral disc and lateral crest, we used shotgun proteomics to identify proteins in a preparation of isolated ventral discs. Candidate disc-associated proteins, or DAPs, were GFP-tagged using a ligation-independent high-throughput cloning method. Based on disc localization, we identified eighteen novel DAPs, which more than doubles the number of known disc-associated proteins. Ten of the novel DAPs are associated with the lateral crest or outer edge of the disc, and are the first confirmed components of this structure. Using Fluorescence Recovery After Photobleaching (FRAP) with representative novel DAP::GFP strains we found that the newly identified DAPs tested did not recover after photobleaching and are therefore structural components of the ventral disc or lateral crest. Functional analyses of the novel DAPs will be central toward understanding the mechanism of ventral disc-mediated attachment and the mechanism of disc biogenesis during cell division. Since attachment of *Giardia* to the intestine via the ventral disc is essential for pathogenesis, it is possible that some proteins comprising the disc could be potential drug targets if their loss or disruption interfered with disc biogenesis or function, preventing attachment.

## Author Summary

*Giardia* is a unicellular intestinal parasite that infects millions of people worldwide each year. Colonization of the small intestine is a critical stage in *Giardia* infection. *Giardia* colonizes the intestinal wall using a specialized suction cup-like structure, the ventral disc. Little is known about the protein composition of the disc or about how the disc functions during attachment. We identified and confirmed eighteen new ventral disc proteins in a preparation of isolated discs using modern genomic methods for analyzing protein composition. We imaged these disc proteins in *Giardia* cells by labeling the proteins with fluorescent tags. A number of these proteins were present on the rim of the ventral disc, a region that appears necessary for the disc to form a seal during attachment to the host. These new ventral disc proteins form the building blocks of the ventral disc structure. Future studies of the roles of the ventral disc proteins either in the assembly of the ventral disc during cell division, or in the functioning of the disc during attachment will enable a better understanding of *Giardia*'s colonization of the host.

## Introduction

*Giardia intestinalis* is a widespread zoonotic parasitic protist. Infection with this parasite results in giardiasis, a common protozoan intestinal disease. Both chronic and acute giardiasis contribute to high morbidity rates in developed [1] and developing countries [2]. Due to a continuing lack of concerted research efforts into the basic biology and mechanisms of pathogenesis of *Giardia*, giardiasis has been designated a World Health Organization (WHO) neglected disease [2]. The growing need for identification of alternative anti-giardial compounds is underscored by recent evidence of resistance to the widely used anti-giardial drug metronidazole [3], [4], [5].

*Giardia* has a two-stage life cycle characterized by an infectious “cyst” form that persists in the environment [6], [7] and a flagellated “trophozoite” form that colonizes the small intestine, causing the characteristic symptoms of giardiasis. Attachment is essential for pathogenesis [8]. Giardiasis remains a serious concern worldwide in areas that lack proper sanitation because of contamination of potable water by giardial cysts [9]. When ingested, giardial cysts begin to “excyst” in the stomachs of their mammalian hosts. In the small intestine, motile trophozoites attach non-invasively and colonize the intestinal epithelium using a specialized cytoskeletal organelle termed the ventral disc [10]. Unattached trophozoites enter the large intestine, “encyst”, and are eventually passed on into the environment.

To proliferate and colonize the small intestine of the host, trophozoites find suitable sites for attachment using flagellar motility [11], and must then remain attached to avoid peristalsis. Giardial attachment via the ventral disc, either to biological surfaces or to inert laboratory surfaces such as culture tubes or slides, is a rapid, stepwise process that occurs in seconds [12]. We have recently shown that flagellar motility is not directly required to maintain attachment [12], invalidating the most widely held model of giardial attachment, the “hydrodynamic model” [13]. Alternative mechanisms for giardial attachment could include overall conformational changes in the ventral disc that could be directly or indirectly responsible for attachment to surfaces. These disc conformational changes could be sufficient to generate suction for *in vitro* attachment or could result in “grasping” of the intestinal epithelium *in vivo*. Alternatively, the rigid structure of the ventral disc may indirectly contribute to attachment by maintaining a negative pressure differential underneath the disc that is created by some other unknown mechanism [10], [14], [15]. Conflicting biophysical data [13]–[20], and a lack of knowledge of molecular components comprising the ventral disc [7] have made it challenging to evaluate any proposed attachment mechanism at the molecular level.

The ventral disc is a highly ordered and complex spiral microtubule array (∼150–400 nm thick) with elaborated structures that protrude dorsally into the cell body [10], [21]–[25]. The “bare area” region, lacking MTs, is located in the center of the array, ventral to the flagellar basal bodies [7]; this structure contains numerous membrane-bound vacuoles [10]. The ventral disc is comprised of three primary structural elements: 1) a right-handed spiral sheet of uniformly spaced MTs (∼250–300 nm apart); 2) trilaminar “microribbons” extending dorsally along the entire length of the MT spiral [24], [25]; and 3) regularly spaced “crossbridge” structures linking adjacent microribbons [24]. The ventral disc MT spiral is physically linked to the ventral plasma membrane by small MT-associated structures termed “sidearms” [24]. The composition and function of the trilaminar microribbons, microribbon-connecting crossbridges, and MT-associated sidearm structures are unknown. The periphery of the ventral disc is surrounded by another highly ordered structure of unknown composition, the lateral crest [26], which has purported, yet unconfirmed, contractile function [21]. We have recently shown using Total Internal Reflection Fluorescence Microscopy (TIRFM) that the lateral crest region contacts the attachment surface, forming a seal during attachment [12]. Finally, a partial left-handed MT spiral array, the supernumerary MT array, lies either dorsal or ventral to the main ventral disc structure and may also possess partially-formed microribbons [24]. The function of the supernumerary MTs in attachment or disc biogenesis is unknown. In summary, the ventral disc MT spiral with associated microribbons and sidearms, the lateral crest, and the supernumerary MTs all comprise the complex structure of the ventral disc required for giardial attachment [27].

Disc-associated proteins were initially termed “giardins”. Three separate gene families of giardins are now known to localize to the ventral disc: three annexins, or alpha-giardins [28]–[31]; three striated fiber (SF)–assemblins, including beta-giardin, delta-giardin, and SALP-1 [32]; and one novel protein, gamma-giardin [33]. Several disc-associated proteins have cell cycle-specific localization, including an ERK1 kinase homolog that localizes to the disc during encystation [34]. Recently, aurora kinase was shown to localize to the ventral disc during cell division [35], yet the localization of aurora kinase to specific structural elements within the disc, and its role, if any, in interphase remains unknown. Two other putatively cell cycle-specific disc-associated Nek kinases were recently identified in a screen for basal body-associated proteins [36]. Thus, while fifteen proteins are now known to localize to the ventral disc at some point in the cell cycle [28]–[36], the composition of each of the primary ventral disc structures (e.g., microribbons, crossbridges, lateral crest) remains to be determined.

Here we used a “shotgun” proteomic strategy [37] with a detergent-extracted, isolated ventral disc preparation to discover novel ventral disc and lateral crest proteins. Candidate disc-associated proteins (or “DAPs”) were identified through peptide sequence analysis and comparisons to the completed *Giardia* genome [38]. Candidate DAPs were then verified by construction of C-terminal DAP::GFP fusions using a high throughput cloning strategy we recently modified for use in *Giardia* [39]. Transformation of *Giardia* with the GFP fusion constructs allowed us to assess the localization of over 50 putative DAPs, and to confirm previously identified [32], [33], [40] and novel DAPs using GFP-tagging and live imaging of GFP fusion proteins in trophozoites. Live imaging of GFP-tagged DAPs also enabled us to distinguish between stable and dynamic pools of representative DAPs using Fluorescence Recovery After Photobleaching (FRAP) [39]. Ultimately, functional analyses of these novel structural DAPs and of any as-yet-unidentified, but potentially dynamic or regulatory, DAPs will be central toward understanding the mechanism of ventral disc-mediated attachment and testing attachment hypotheses.

## Materials and Methods

### Strains and culture conditions

*Giardia intestinalis* strain WBC6 (ATCC 50803) trophozoites were maintained at 37°C in modified TYI-S-33 medium with bovine bile [41] in 16 ml screw cap tubes (Fisher Scientific).

### Detergent extraction of ventral discs for proteomic analysis

**Figure 1:**
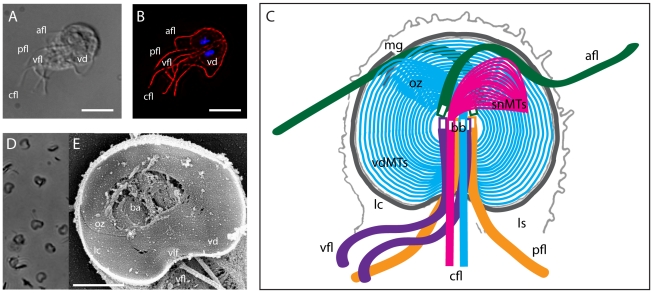
Structure of the cytoskeleton and ventral disc in Giardia. Panels A (DIC) and B (anti-alpha-tubulin immunostaining) illustrate the primary microtubule structures of interphase trophozoites including the ventral disc (vd), and the four pair of flagella: anterior (afl), ventral (vfl), posteriolateral (pfl) and caudal (cfl). Scale = 5 µm. The structures of the ventral disc and flagellar basal bodies are illustrated in the schematic in panel C, including the ventral disc microtubule (MT) array (vdMTs) and the lateral crest (lc), the overlap zone of the ventral disc MT spiral (oz), the supernumerary MT array (snMTs), and the four pair of flagella and eight basal bodies (bb). Other areas of the ventral side of the giardial cell, including the lateral shield (ls) and the marginal groove (mg) are also shown. The detergent-extracted disc preparation used for proteomic analysis is shown in panel D (phase contrast), and a similarly extracted cytoskeletal preparation is shown in panel E. The ventral disc (vd), ventrolateral flange (vlf), lateral crest (lc) and overlap zone (oz) are shown, as well as the bare area (ba) and underlying flagellar basal bodies (SEM courtesy of Joel Mancuso). Scale = 2 **µ** ***m***.

The primary goal in the isolation of intact ventral discs for proteomic analysis was the maintenance of microtubule-associated proteins, by removing radicals and metal ions that could damage disc structure, and by stabilizing microtubules using drugs like Taxol. We modified a cytoskeletal preparation from Holberton et al. [42] to isolate disc and flagellar cytoskeletons from *Giardia*. First, TYI-S-33 medium was decanted from one confluent 12 ml culture of trophozoites. Cells were demembranated and cytoskeletons were extracted by adding 1 ml of 1% Triton X-100 in 1X PHEM plus Taxol (60 mM PIPES, 25 mM HEPES, 10 mM EGTA, 1 mM MgCl_2_, pH 7.4, 1 mM DTT, 10 µM paclitaxel (Sigma)) and vortexing continuously at the highest setting for 3 minutes. To prevent proteolysis, protease inhibitors (Roche) were added to the preparation. Ventral disc cytoskeletons were then pelleted by centrifugation at 16,000×g, and the pellets were washed four times in 1X PHEM+Taxol lacking 1% Triton X-100. Sufficient extraction of cytoskeletons was confirmed by wet mount using phase contrast or DIC microscopy (see Figure 1).

### Proteomic analysis of a detergent extracted ventral disc preparation

We identified the proteins present in the ventral disc preparation using liquid chromatography tandem mass spectrometry (LC-MS/MS LTQ) [37]. All MS/MS samples were analyzed using X! Tandem (www.thegpm.org; version TORNADO (2008.02.01.2)). X! Tandem was set up to search protein sequences downloaded from Genbank (*Giardia intestinalis*) assuming the digestion enzyme trypsin. X! Tandem was searched with a fragment ion mass tolerance of 0.40 Da and a parent ion tolerance of 1.8 Da. Iodoacetamide derivative of cysteine was specified in X! Tandem as a fixed modification. Deamidation of asparagine, oxidation of methionine, sulphone of methionine, tryptophan oxidation to formylkynurenin of tryptophan and acetylation of the N-terminus were specified in X! Tandem as variable modifications.

Scaffold (version Scaffold_2_03_01, Proteome Software Inc., Portland, OR) was used to validate MS/MS based peptide and protein identifications. Peptide identifications were accepted if they could be established at greater than 80.0% probability as specified by the Peptide Prophet algorithm [43]. Protein identifications were accepted if they could be established at greater than 95.0% probability and contained at least one identified peptide. Protein probabilities were assigned by the Protein Prophet algorithm [44]. Proteins that contained similar peptides and could not be differentiated based on MS/MS analysis alone were grouped to satisfy the principles of parsimony.

### C-terminal GFP-tagging of candidate disc-associated proteins (DAPs) using Gateway cloning

Fifty-eight of the 102 candidate DAPs identified in the proteomic survey were chosen for localization; candidates that appeared to be metabolic, flagellar-associated or chromatin-associated proteins were excluded. All candidate DAP PCR forward primers (see Table S1) were designed to bind approximately 200–250 bp upstream of the gene to include the *Giardia* native promoter and contained the sequence CACC at the 5′ end to facilitate directional cloning. Blunt-ended PCR amplicons were generated by PCR using *PfuTurbo* Hotstart PCR Mastermix (Stratagene) with *Giardia intestinalis* strain WBC6 genomic DNA. The candidate DAP PCR amplicons were subsequently subcloned into the Invitrogen pENTR/D-TOPO backbone to generate Gateway entry clones. Inserts in entry clones were sequenced to confirm the identity and correct orientation of the gene. To construct DAP::GFP fusions, positive entry clones were then recombined, via LR reaction, with a 1-fragment GFP tagging *E. coli*/*Giardia* shuttle destination vector (pcGFP1F.pac, [39]) using LR Clonase II Plus (Invitrogen). LR reactions were performed using 100 ng pcGFP1F.pac and 150 ng of DAP entry clone plasmid DNA. Positive clones were screened by digestion with *Asc*I, and bulk plasmid DNA was prepared using Qiagen's Endofree Plasmid Maxi Kit.

To create C-terminal GFP-tagged candidate DAP strains, *Giardia intestinalis* strain WBC6 was electroporated with roughly 20 µg of plasmid DNA (above) using the GenePulserXL (BioRad) under previously described conditions [45]. Episomal DAP::GFP constructs were maintained in transformants using antibiotic selection (50 µg/ml puromycin) [46].

### Immunofluorescence microscopy and image data analysis

Immunostaining of the GFP-tagged DAP strains was performed as previously described [45]. To confirm disc localization, Metamorph image acquisition software (MDS Technologies) was used to collect 3D images using a Leica DMI 6000 wide-field inverted fluorescence microscope with a PlanApo 100X, NA 1.40 oil immersion objective. Serial sections of DAP::GFP strains were acquired at 0.2 µm intervals, and deconvolved using Huygens Professional deconvolution software (SVI). Two dimensional maximum intensity projections were created from the 3D data sets for presentation purposes.

### Assessment of DAP::GFP fusion protein turnover using FRAP

We used laser fluorescence photobleaching of specific regions to measure the movement and steady state turnover of the new DAPs in *Giardia*, a technique that has been used extensively in other organisms [47]. Three DAP::GFP-expressing strains (whole disc, DAP5374; lateral crest, DAP13981; and disc plus axonemes, DAP17090) were selected as representative examples of different ventral disc localizations. The media in a confluent 12 ml culture was replaced with 1X HBS for 1 hour at 37°C. The culture was then iced for 15 minutes to detach cells, and 3 ml of the cell suspension were transferred to a coverslip placed in an 8-well plastic plate. Cells were incubated for 30 minutes at 37°C under nitrogen gas to allow them to attach to the coverslip. After 1 hour, 1 µl of CellMask orange (Invitrogen) was added to the cell suspension. Stained cells were incubated for 5 minutes at 37°C then rinsed twice with warmed 1X HBS. The edges of the coverslip were blotted and the coverslip was inverted onto a slide with double stick tape. Warmed 2% low-melt agarose (Sigma) in 1X HBS was added under the coverslip to embed the attached cells and the prep was sealed on all sides with VALAP. An Olympus FV1000 scanning laser confocal microscope equipped with a four channel PMT was used for imaging and simultaneous 405 nm bleaching. The pre-bleach image of the cell was acquired using a 60×, 1.42 NA objective and a 488 nm laser (at 5% with 4 µs/pixel scan speed). To photobleach a specific region of DAP localization, we used the 405 nm laser (90% power for 200 milliseconds). Fluorescence recovery in the GFP-tagged DAP strains was assessed by imaging once every minute, for up to 10 minutes, using the 488 nm low power laser excitation. Normalized GFP fluorescence recovery was calculated by subtracting the PMT background noise from the ROI intensity measurement; the background-subtracted intensity measurement was then divided by a fluorescent control ROI intensity measurement to normalize for photobleaching due to imaging.

### Negative staining and immunolabeling of isolated discs from DAP::GFP fusions

Detergent extracted cytoskeletons containing GFP-tagged DAPs were isolated (see above). Immunolabeling and negative staining of the isolated discs was performed as previously described [48] in 1.5 ml Eppendorf tubes with gentle shaking at room temperature. Cytoskeletons were placed in a blocking buffer of 3% nonfat dry milk in PHEM buffer (60 mM PIPES, 25 mM HEPES, 10 mM EGTA, 2 mM MgCl_2_) for 1 hour. Cytoskeletons were then labeled with an anti-GFP antibody in blocking buffer for 1.5 hours, and then rinsed 3 times, for 15 minutes each, in PHEM. Pelleting between steps was done at 2,000×g for 5 minutes with a short vortexing step for resuspension. Samples were incubated with 5 nm goat-anti-rabbit F(ab′)2 IgG antibody (BB International) in blocking buffer for 1 hour, then rinsed 3 times in PHEM for 15 minutes. For negative controls (not shown), we used secondary antibody only.

For negative staining of DAP::GFP fusion strains, 300 mesh copper grids (EMS) were Formvar-coated, carbon-coated, then glow-discharged to make them more hydrophilic. A 5 µl droplet of cytoskeleton solution was placed on the grid, blotted, and then negative stained with a 5 µl droplet of 2% aqueous uranyl acetate (Ted Pella) and blotted. Grids were imaged with an AMT digital camera in a CM100 (FEI) transmission electron microscope operating at 80 kV.

## Results

### Discovery of novel components of the ventral disc and lateral crest

**Figure 2:**
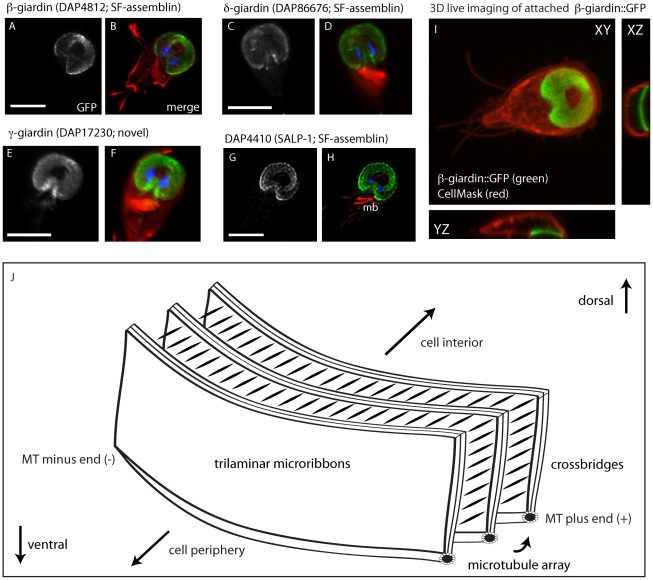
GFP tagging of known disc-associated proteins does not affect subcellular localization, disc conformation, or attachment. Panels A–H show the ventral disc localization of C-terminal GFP-tagged, previously described disc-associated proteins beta-giardin (A,B), delta-giardin (C,D), gamma-giardin (E,F) and SALP-1 (G,H) (also see Table 1). The dome-shaped structure of the ventral disc is visible using 3D live imaging in a GFP-tagged beta-giardin strain (with the plasma membrane counterstained in red using CellMask (Invitrogen)) (I). Note the curvature of the ventral disc as visualized by live imaging in attached beta-giardin::GFP trophozoites. Scale = 5 µm. A schematic of the structural elements of the ventral disc (J) shows the curved ventral disc microtubules with associated trilaminar microribbons that protrude into the cytoplasm. The crossbridges connecting the microribbons are also shown.

The detergent extracted disc preparation for proteomic analysis yielded 102 candidate disc-associated proteins. The list of candidate disc proteins and their GiardiaDB (www.giardiadb.org) accession numbers are shown in Table S2. Based on protein functional predictions and motif analysis, we classified these proteins into three categories: putatively disc-associated (57 total), putatively flagellar (17 total), or metabolic or chromatin-associated (28 total). Flagellar proteins were likely present due to the presence of axonemes in the disc preparation; only a few were confirmed to localize to the flagella by GFP-tagging (Table S2). Metabolic or chromatin-associated proteins were deemed contaminants unlikely to be associated with the ventral disc. We confirmed the presence and localization of known disc-associated proteins identified by our proteomic analysis (Figure 2 and Table 1), including beta-giardin [22], delta-giardin [40], gamma-giardin [33] and SALP-1 [32]. We also identified three annexins (alpha-2, alpha-6, and alpha-17) in the disc proteome. We have previously shown that alpha-2 annexin localizes to the ventral disc and ventral flagella [12]. We did not identify several annexins [28], aurora kinase [35], or several other proteins previously reported to localize to the ventral disc [34], [36], [49], possibly due to slight differences in sample preparation.

**Table 1:** Known and novel disc-associated proteins.

GiardiaDB	GiardiaDB	GiardiaDB								
A (WBC6)	B (GS)	E (P15)	Name	Size (aa)	MW (kDa)	Motifs	InterPro	Localization	Method	References
GL50803_112079	GL50581_1380	GLP15_2255	alpha-2-tubulin	454	51	tubulin	PF00091	disc, MTs	het. Ab	[23]
GL50803_101291	nf	GLP15_1773	beta tubulin	447	50	tubulin	PF00091	disc, MTs	het. Ab; GFP	[23]; This study
GL50803_7796	GL50581_1672	GLP15_898	alpha-2 annexin	296	34	annexin	SSF47874	VFL or disc	AU1; GFP	[27]; [12]
GL50803_11683	GL50581_4264	GLP15_3639	alpha-3 annexin	296	33	annexin	SSF47874	disc	AU1	[28]
GL50803_7797	GL50581_1671	GLP15_899	alpha-5 annexin	302	34	annexin	SSF47874	diffuse disc, VFL	AU1	[28]
GL50803_15101	GL50581_2463	GLP15_4751	alpha-17 annexin	310	35	annexin	SSF47874	disc, VFL or weak cyto	AU1; GFP	[28]; This study
GL50803_4812	GL50581_2741	GLP15_2766	Beta-giardin	272	31	SF-assemblin	PF06705	disc	*Giardia* Ab; GFP	[22]; This study
GL50803_86676	GL50581_3174	GLP15_2196	Delta-giardin	293	31	SF-assemblin	PF06705	disc	*Giardia* Ab; GFP	[40]; This study
GL50803_17230	GL50581_4532	GLP15_1116	Gamma-giardin	311	36	none	none	disc	*Giardia* Ab; GFP	[33]; This study
GL50803_4410	GL50581_1154	GLP15_3863	SALP-1	255	30	none	none	disc	AU1; GFP	[32]; This study
GL50803_5358	GL50581_494	GLP15_3713	aurora kinase	311	36	protein kinase	SSF56112	disc during cytokinesis	het. Ab	[35]
GL50803_17563	GL50581_1780	GLP15_2679	ERK1 kinase	385	44	protein kinase	SSF56112	disc edge, BB, MB, CFL	*Giardia* Ab	[34]
GL50803_16279	GL50581_694	GLP15_1761	Nek kinase	661	75	protein kinase	SSF56112	disc	AU1	[36]
GL50803_92498	GL50581_75	GLP15_1443	Nek kinase	898	102	protein kinase	SSF56112	faint disc, cyto axonemes, MB	AU1	[36]
GL50803_5010	GL50581_2811	GLP15_4970	Ser/Thr Phosphatase PP2A-2 subunit	344	344	metallo-dependent phosphatase	SSF56300	disc, cyto AFL, PFL and CFL, BB	het. Ab	[40]
GL50803_103807	GL50581_4392	GLP15_4033	ankyrin repeat protein	946	103	ankyrin repeat	SSF48403	disc	GFP	This study
GL50803_17053	GL50581_1272	GLP15_3594	ankyrin repeat protein	1044	114	ankyrin repeat	SSF48403	disc, except VLF	GFP	This study
GL50803_16343	GL50581_686	GLP15_1754	MB protein	857	101	none	none	disc, MB	GFP; *Giardia* Ab	This study; [54]
GL50803_24321	GL50581_3117	GLP15_3007	Nek kinase	288	33	protein kinase	SSF56112	disc, especially posterior tips, cyto	GFP	This study
GL50803_5374	GL50581_2499	GLP15_1088	CAP_GLY protein	239	27	CAP_Gly; UBQ	SSF54236	disc, VLF	GFP	This study
GL50803_13766	GL50581_186	GLP15_925	ankyrin repeat protein	843	95	ankyrin repeat	SSF46966	disc	GFP	This study
GL50803_14872	GL50581_1443	GLP15_4117	ankyrin repeat protein	1251	137	ankyrin repeat	SSF48403	faint disc edge	GFP	This study
GL50803_17231	GL50581_4533	GLP15_1115	Nek kinase/ankyrin repeat protein	1006	111	protein kinase; ankyrin repeat	SM00220	disc edge	GFP	This study
GL50803_23492	GL50581_1458	GLP15_4102	ankyrin repeat protein	743	80	ankyrin repeat	SSF48403	disc edge	GFP	This study
GL50803_24194	GL50581_2322	GLP15_2357	ankyrin repeat protein	786	86	ankyrin repeat	SSF48403	disc edge	GFP	This study
GL50803_103810	GL50581_4395	GLP15_4036	ankyrin repeat protein	1873	206	ankyrin repeat	SSF48403	disc edge, BA edge	GFP	This study
GL50803_17096	GL50581_1574	GLP15_4933	ankyrin repeat protein	760	85	ankyrin repeat	SSF48403	disc edge, BB	GFP	This study
GL50803_17097	GL50581_1573	GLP15_4934	ankyrin repeat protein	1457	163	ankyrin repeat	SSF48403	disc edge, BB, cyto AFL; MB and cyto CFL	GFP	This study
GL50803_16424	GL50581_2872	GLP15_4873	hypothetical protein	252	30	none	none	punctate disc edge, BB, cyto AFL	GFP	This study
GL50803_13981	GL50581_2633	GLP15_1140	Nek kinase/ankyrin repeat protein	1109	123	protein kinase; ankyrin repeat	SSF56112	disc edge, VLF	GFP	This study
GL50803_15576	GL50581_1505	GLP15_2307	ankyrin repeat protein	750	82	ankyrin repeat	SSF48403	posterior disc edge, VLF	GFP	This study
GL50803_17090	GL50581_1587	GLP15_2758	SAM motif protein	181	21	SAM	SSF44769	snMTs, BB, flagella	GFP	This study
GL50803_16263	GL50581_1383	GLP15_2420	DIP13	106	12	none	none	snMTs, BB, flagella	GFP	This study

Previously described and novel disc-associated proteins identified in this study are summarized with protein domain information and GFP localization (nf = not found, het Ab = heterologous antibody, cyto = cytoplasmic, MTs = microtubules, snMTs = supernumerary MTs, BB = basal bodies, MB = median body, BA = bare area, VLF = ventrolateral flange, AFL = anterior flagella, CFL = caudal flagella, VFL = ventral flagella, and PFL = posteriolateral flagella).

**Figure 3:**
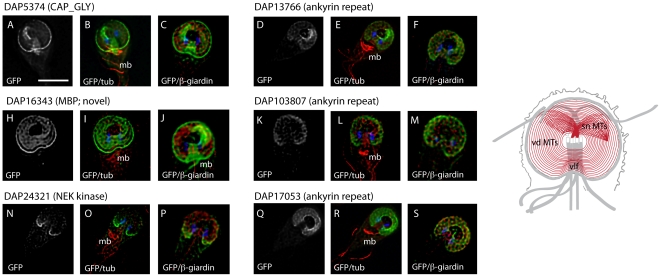
Novel disc-associated proteins associated with the entire disc. Six new DAPs localize to the entire ventral disc spiral in a manner similar to the previously described microribbon-associated proteins (see Figure 2 and Figure S3) as visualized by C-terminal GFP tagging (grey or green) and either anti-alpha-tubulin immunostaining of the MT cytoskeleton (red), or anti-beta-giardin immunostaining of the ventral disc microribbons (red). The two nuclei are visible with DAPI staining (blue). One putatively microtubule-associated DAP possesses a CAP-Gly motif (A,B,C). Another disc-localizing DAP is the possibly mis-named median body protein (MBP, H,I,J). Three ventral disc-localizing DAPs have ankyrin repeat domains (D–F, K–M, and Q–S). Note the absence of GFP localization in the ventrolateral flange (vlf) area in DAP17053 (Q–S). Finally, one disc-localizing DAP is a Nek kinase that has a greater localization to the posterior region of the ventral disc (N–P). Scale = 5 µm. Schematic shows areas of GFP localization in red.

**Figure 4 pntd-0001442-g004:**
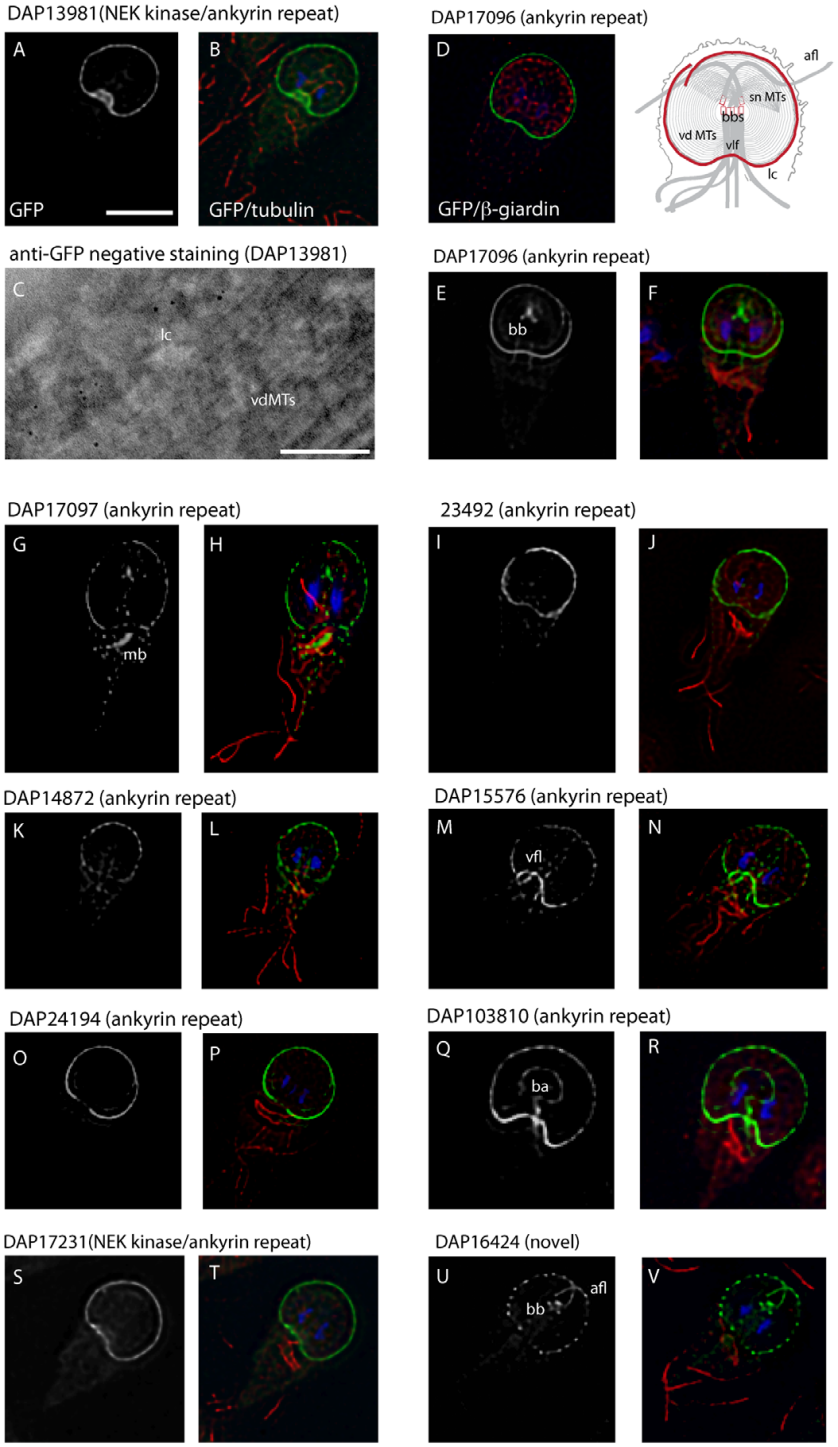
A diversity of disc-associated proteins localize to the lateral crest. Ten new DAPs localize to the lateral crest (lc) structure that surrounds the ventral disc (see red, schematic) and comes in close contact with the intestinal epithelium during attachment [12]. In all immunostained images except (D), DAPs are visualized using C-terminal GFP tagging (grey or green) in trophozoites counterstained with anti-alpha-tubulin immunostaining of the MT cytoskeleton (red) and DAPI staining of the two nuclei (blue). DAP13981 (A,B) is an ankyrin repeat-containing Nek kinase that localizes clearly to the lateral crest when imaged using anti-GFP immunogold labeling and negative staining (C); scale = 200 nm. In (D), DAP17096::GFP localization (green) is shown in a trophozoite immunostained with anti-beta-giardin (red) to highlight the ventral disc microribbons, emphasizing the lack of colocalization of the lateral crest proteins with the main structure of the ventral disc. In some cases, lateral crest-localizing DAPs associate with other structures, such as the basal bodies (bb) (E-H,U,V and see Figure 6) and the inner region of the ventral disc surrounding the bare area (ba) (DAP103810; Q,R). All of the lateral crest localizing-DAPs contain ankyrin repeats except DAP16424 (U,V), a novel protein that has a punctate localization to the lateral crest and the cytoplasmic region of the anterior axonemes (afl) and basal bodies (bb). Scale for immunostained images = 5 µm. Schematic shows areas of GFP localization in red.

**Figure 5:**
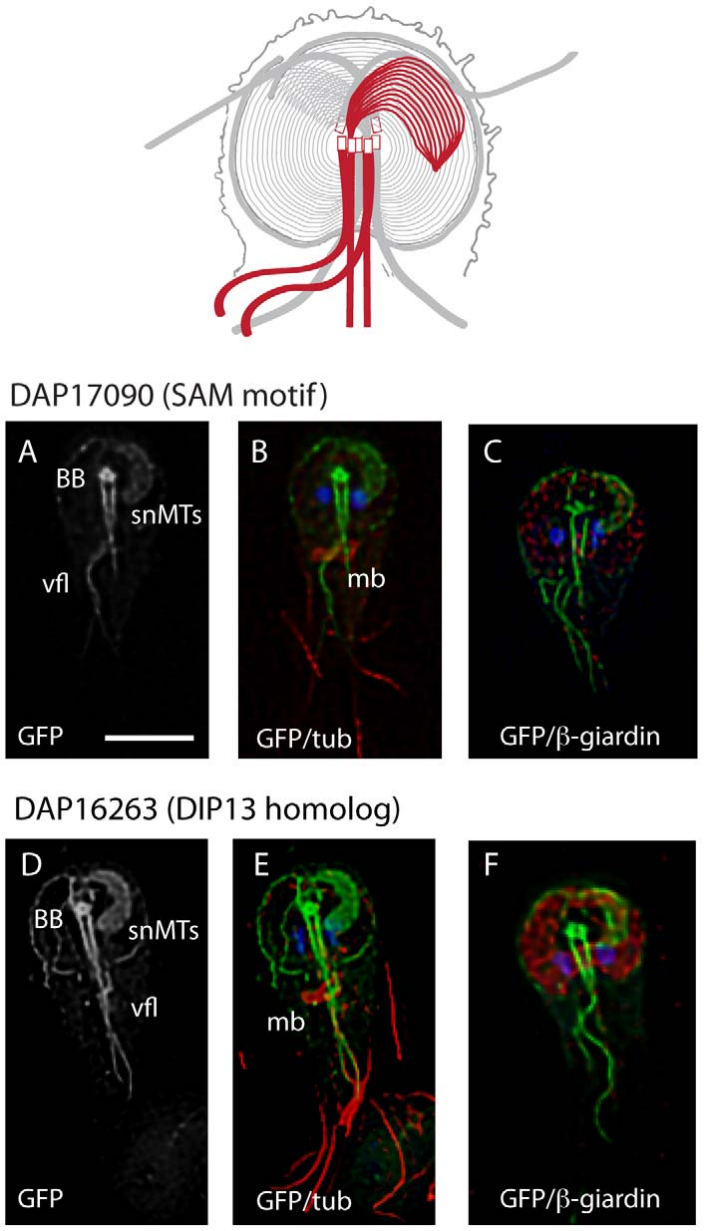
*DAP17090::GFP and DAP16263::GFP localize to the supernumerary microtubules and axonemes*. Both DAP17090 (A–C), which contains a SAM protein-protein interaction motif, and DAP16263 (D–F), a DIP13 microtubule binding protein homolog, localize to the supernumerary MTs that are slightly dorsal or ventral to the main ventral disc microtubules (for 3D stacks, see Video S1 and Video S2). These DAPs also have a transient (see Figure 6) localization to the basal bodies (bb) and the caudal (cfl) and ventral (vfl) flagella. DAPs are again visualized using C-terminal GFP tagging (grey or green) in cells counterstained with anti-alpha-tubulin immunostaining of the MT cytoskeleton (red) and DAPI staining of the two nuclei (blue). Scale = 5 µm. Schematic shows areas of GFP localization in red.

We created C-terminal GFP fusions to 58 giardial proteins using a high-throughput Gateway-based cloning strategy for *Giardia* [39] followed by transformation into trophozoites (see Materials and Methods). Ventral disc or lateral crest localization was confirmed for 18 novel DAPs of the 57 candidates using 3D deconvolution microcopy (Table 1, and Figure 3, Figure 4 and Figure 5). Candidate proteins that were found to localize to the ventral disc were not necessarily those for which the greatest number of mass spectra were obtained (Table S2); of the 18 new DAPs, a relatively large number of spectra was obtained only for DAP16343. Non-disc localizations included the basal bodies, the median body, regions of various flagellar pairs, or the cytoplasm. We also noted a lack of GFP expression in some interphase trophozoites (Table S2), which could indicate cell cycle-specific expression.

Each confirmed DAP has a conserved homolog in the other two sequenced *Giardia* genomes [50], [51]. Two novel disc-associated proteins (DAP16424 and DAP16343) had no conserved motifs or known homologs in other organisms. Many other DAPs had conserved motifs including: ankyrin repeats (13 DAPs), Nek kinase domains (3 DAPs), and SAM domains (DAP17090) (see Table 1); some had more than one of these motifs. The disc-associated kinases (DAP24321, DAP17231, and DAP13981) may be non-functional pseudokinases [52], as they lack highly conserved catalytic residues (Figure S1). Two of the newly identified DAPs are putatively microtubule-associated, including DAP5374, a CAP-Gly motif containing protein [53], and DAP16263, a DIP13 homolog [54] with a conserved MT-binding motif (see Figure S2). Finally, we observed that the “median body protein” (DAP16343) [55] has an obvious disc localization with occasional localization to the median body (Figure 3). We categorized the eighteen novel disc associated proteins into three general types of localization: to the entire disc spiral (Figure 3), to the disc edge or lateral crest (Figure 4), or to the supernumerary MTs (Figure 5). In some cases, we observed additional localization to the basal bodies, to regions of the axonemes (Figure 5), or to the median body (Figures 2 and 3).

### Six novel DAPs localize to the entire spiral of the ventral disc

Eight ventral disc-associated proteins have previously been localized using specific antibodies. We show that GFP-tagged DAP localization concurs with the localizations previously described using immunostaining (Table 1 and Figure 2) with the exception of alpha17-annexin (Table S2). We confirmed localization to the entire ventral disc spiral for the SF-assemblin homologs (e.g., beta-giardin, delta-giardin, and SALP-1) and gamma-giardin (Figure 2). Notably, we also find some localization of these DAPs to the median body, primarily during prophase. Using anti-GFP immunogold labeling with negative staining [25], we demonstrate that the SF-assemblin homologs and gamma giardin localize to the microribbons [22] that are bound to the spiral microtubule array and project into the cytoplasm (Figure S3).

Six novel disc-associated proteins localized to the entire disc (Figure 3), including three ankyrin repeat proteins (DAP13766, DAP103807 and DAP17053), the median body protein (MBP; DAP16343), one CAP-Gly protein (DAP5374) and one Nek kinase (DAP24321). The ankyrin repeat protein DAP17053 has a unique localization in that it is present in throughout the ventral disc spiral, but is completely absent in the ventrolateral flange region (see Figure 3). The Nek kinase DAP24321 is present throughout the cell and ventral disc but localizes most strongly to the posterior regions of the disc. DAP16343 (MBP) localizes throughout the disc spiral and the lateral crest, and DAP5374 localizes throughout the disc spiral and the lateral crest.

### Many DAPs localize to the disc edge and are presumptive components of the lateral crest

The ventral disc spiral is surrounded by a putatively contractile repetitive structure termed the “lateral crest” (Figure 1 and [26]). Ten of the novel DAPs localize primarily to the disc perimeter, presumptively in the region of the lateral crest or along the outside edge of the ventral disc spiral (Figure 4). Two (DAP13981 and DAP17231) are Nek kinases with ankyrin repeat domains, another (DAP16424) is novel with no homology to known proteins or domains, and the remaining seven are ankyrin repeat proteins (Table 1 and Figure 4). The Nek kinase DAP13981 was specifically localized to the lateral crest using negative staining with anti-GFP immunogold labeling (Figure 4). One ankyrin repeat protein (DAP103810) is notable in that it also localizes to the inner perimeter of the disc, near the “bare area” while all others localize only to the outer disc edge. DAP16424 has a regularly spaced, punctate localization around the disc edge, and is also present at the basal bodies and cytoplasmic regions of the anterior axonemes. In addition to localizing to the disc perimeter, DAP17097 also localizes to the median body in many cells. DAP17096, DAP17097, and DAP16424 also localize to the basal bodies during interphase.

### Two DAPs localize to the supernumerary microtubule array

We identified two novel DAPs that localize specifically to the supernumerary MTs (Figure 1) and to axonemes, yet not to the entire ventral disc structure (see schematic in Figure 5). DAP17090, a novel protein containing a SAM motif, localizes to the supernumerary MTs, the ventral flagella axonemes, the cytoplasmic regions of the caudal and anterior axonemes, and to the basal bodies (Figure 5 and Video S1). DAP16263, a DIP13 homolog, has a similar localization and also localizes faintly to the lateral crest (Figure 5 and Video S2).

### DAP::GFP fusion proteins turn over only at non-disc locations such as the axonemes and basal bodies

**Figure 6:**
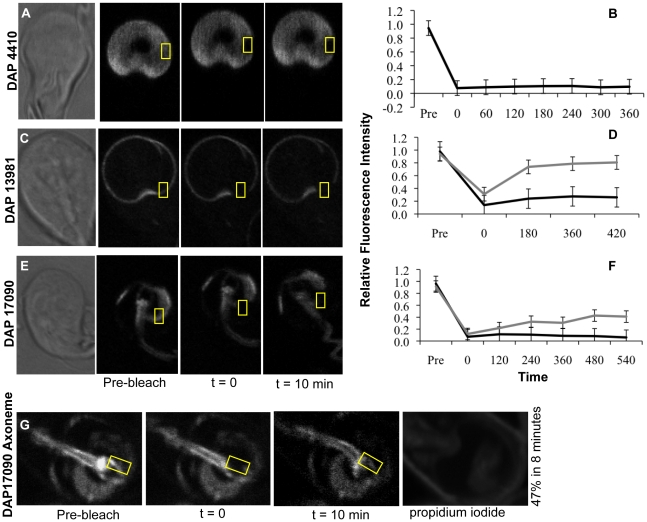
DAPs recover only at non-disc, axoneme or basal body regions. Cells stably transformed with expression plasmids encoding full-length DAP::GFPs were subjected to quantitative FRAP analysis. Panel A shows representative images of DAP4410::GFP, from the group of proteins that localize to the entire disc spiral. No recovery is observed for DAP4410::GFP 10 minutes post-bleaching (B), and no additional non-disc localization was observed throughout the cell cycle. Panel C shows DAP13981::GFP, a representative of the lateral crest-localizing DAPs. DAP13981::GFP does not recover after 10 minutes post-bleach when localized to the lateral crest of the ventral disc (black line, D), but exhibits significant recovery when localized to the basal bodies or axonemes (gray line, D). DAP17090::GFP (E) is a representative supernumerary MT-associated DAP. No recovery of DAP17090 is observed at the supernumerary MTs at 10 minutes post-bleach (black line, F). Panel (G) shows a DAP17090::GFP cell with basal body and axoneme localization of the protein, which partially recovers within 3 minutes post-bleach (gray line, F). Propidium iodide was used to monitor cell viability (G). Relative fluorescence intensity was corrected for overall loss of fluorescence due to imaging and normalized for background. Black lines indicate a disc-associated region of interest (N = 10), whereas gray lines indicate a BB/axoneme recovery region (N = 10).

To assess whether DAPs localizing to the ventral disc are stable or dynamic, we used FRAP to examine protein turnover in representative GFP-tagged DAP strains (for the lateral crest, DAP13981; for the entire disc, DAP4410 (SALP-1); and for the supernumerary MTs, DAP17090). For each of these DAP::GFP fusions, we observed no recovery of GFP fluorescence to the ventral disc in interphase trophozoites for more than 10 minutes post photobleaching (Figure 6). In the DAP17090::GFP strain with localization to the basal bodies and/or axonemes (26%, n = 100) as well as to the disc, GFP fluorescence did recover, but only to the non-disc structures. We noted a partial recovery (47%) of fluorescence at the axonemes of the DAP17090::GFP strain within 8 minutes. Similarly, the axonemes of the lateral crest strain DAP13981::GFP recovered to 80% within 7 minutes (Figure 6). However, DAP13981 localization to the axonemes was visible only in DAP13981::GFP trophozoites undergoing cytokinesis. We did not observe ventral disc recovery for DAP4410, which only localizes to the ventral disc and not to other cytoskeletal elements (Figure 2).

## Discussion

The ventral disc is a unique cytoskeletal structure that is only present in *Giardia* and not in related diplomonads (Figure 1 and [56]). As the ventral disc is critical for parasite attachment to the host, early work characterized the disc architecture at a basic level, defining primary structural elements (e.g., microribbons and crossbridges) and identifying several microribbon-associated proteins (e.g., beta-giardin) [23], [33]. Over the past 40 years, only a handful of disc-associated proteins comprising those structural elements have been identified using biochemical approaches [28]–[36], [40], [49], and recent studies of disc-mediated attachment [13], [14], [57] have lacked a comprehensive analysis of ventral disc and lateral crest composition with respect to ventral disc functioning and biogenesis.

We identified and confirmed the localization of 18 previously unknown disc associated proteins (Figures 3–5). Taken together with prior work, we now estimate over 30 disc-associated proteins, including alpha and beta tubulin. At least twenty structural proteins comprise the ventral disc and at least ten structural proteins comprise the lateral crest. The novel DAPs include those with conserved sequence motifs, as well as those that lack homology to known proteins in any organism. These novel DAPs probably do not represent an exhaustive list of disc-associated proteins; other disc-associated proteins likely remain to be discovered. Nonetheless, this proteomic analysis of structural components of the ventral disc (DAPs) provides a large collection of proteins for future investigations of ventral disc function in attachment, daughter disc assembly during cell division, or disc assembly/disassembly during encystation or excystation.

### Known DAPs are components of the disc microribbons

We confirmed that several previously identified DAPs are associated with the ventral disc microribbons (Figure S3). The microribbons extend from the spiral MT array into the cytoplasm (Figure 2) and consist of two sheets of globular subunits, separated by a fibrous inner core, forming a structure about 25 nm thick [25]. Beta-giardin and the other previously identified SF-assemblin homologs, including delta-giardin and SALP-1 [32] likely form the structural basis of the microribbons upon which other microribbon-associated proteins assemble [26]. We confirmed the microribbon localization of beta-giardin using GFP-tagging and negative staining (Figure S3), and have also confirmed the microribbon association of delta-giardin, SALP-1, and gamma-giardin, a protein that lacks conserved domains and is unique to *Giardia*. Like beta-giardin [39], the microribbon-associated protein SALP-1 (DAP4410) also does not turn over following photobleaching. Thus we believe that the ventral disc is a relatively stable structure. We hypothesize that microribbon-associated proteins likely assemble into the ventral disc prior cell division, and that MTs of the disc do not undergo rapid polymerization dynamics as was previously proposed [58].

### Conserved protein motifs suggest the functional roles of several novel DAPs

Some DAPs associate with the entire ventral disc structure (Figure 3), yet other DAPs localize to specific structural components in other regions of the ventral disc, including the lateral crest, the ventrolateral flange, and the supernumerary MTs (Figure 4–5). A putative role for some of these disc proteins can be inferred from the conserved motifs they contain.

Two of the novel disc-associated proteins, DAP5374 and DAP16263, have microtubule binding motifs. In general, microtubule-associated proteins mediate dynamic processes of microtubules. They include proteins that promote polymerization or depolymerization dynamics, microtubule end- or side-binding proteins, enzymes that modify tubulin, and microtubule motors such as kinesins and dyneins that generate cellular forces. Many of these have been identified in the *Giardia* genome [38]. DAP5374 is a CAP-Gly protein [53] that also contains an N-terminal ubiquitination (UBQ) motif, indicating that it might target the parental ventral disc for cell cycle-specific degradation and disassembly via a proteasome-dependent pathway. CAP-Gly proteins interact with tubulin monomers, dimers, and/or MTs, regulate microtubule dynamics and organization, and are involved in intracellular signaling and the distribution of cellular organelles.

DAP16263 is a homolog of DIP13, a 13 kDa *Chlamydomonas* protein that defines a new, and likely ancient, MT-associated protein family conserved in diverse protists, plants, and animals that have flagellated cell stages [54], [59]. In *Chlamydomonas*, DIP13 localizes to the centrioles and to cytoplasmic and flagellar MTs, and is purported to either stabilize or connect MTs to other cellular structures [54]. DIP13 homologs contain a conserved MT binding motif – “KREE” – that directly binds MTs [54]. Because the giardial DIP13 homolog (DAP16263) lacks this motif (see Figure S2), it is unclear whether DAP16263 can directly bind MTs. Additionally, antisense RNA knockdown of DIP13 in *Chlamydomonas* results in severe cytological and cell division defects, including improper flagellar number and orientation. We observed localization of DAP16263 to the caudal and the ventral flagella, as well as to the basal bodies and the supernumerary MT spiral. As many components of the ventral disc are MT-associated proteins (CAP-Gly or DIP13 domains) or are related to flagellar root structures (SF-assemblins [60]), the ventral disc may be derived from ancestral flagellar structures.

Several DAPs are NIMA-Related Kinases (Neks). These kinases are ancient members of the large serine/threonine kinase family with putative roles in the cell cycle and in ciliary function [61]. Over seventy Nek kinases are present in the *Giardia* genome [62]. We identified one that localizes to the ventral disc (DAP24321) and two that localize to the lateral crest (DAP13981 and DAP17231). These disc-associated Nek kinases appear to be pseudokinases as they lack conserved catalytic residues ([52] and Figure S1). Two other giardial Nek kinases were also shown to localize to the ventral disc in a recent survey of giardial basal body-associated proteins [36]. Nek pseudokinases could simply contribute to disc and lateral crest structure; however, the lack of functional catalytic sites in pseudokinases does not always result in a lack of kinase activity [63], and some pseudokinases have been assigned roles in the regulation of other kinases [52]. Thus, disc-associated Neks lacking functional catalytic sites might still perform regulatory functions required for attachment dynamics, dorsal daughter disc biogenesis or parental disc disassembly during cell division [64].

Many disc-associated proteins (Table 1) possess conserved ankyrin repeat domains, roughly 33 amino acid protein-protein interaction motifs often present in tandem arrays in diverse proteins in diverse eukaryotes [65]. Ankyrin repeat domain-containing proteins are very abundant (up to 3.6% of ORFs) in the *Giardia* genome [38], [66]. Ankyrin repeat domain-containing DAPs could interact with the microtubules or tubulin [67], could be associated with the microribbons, crossbridges or sidearm structures, or possibly, may connect the ventral disc structure to other cytoskeletal or membrane proteins.

Finally, one intriguing protein of the disc proteome that lacks homology to known proteins is the 101 kDa “median body protein”, or MBP (DAP16343). MBP is clearly an abundant disc protein that may have localization to the median body [68] at specific points in the cell cycle, especially prior to mitosis. Beta-giardin, gamma giardin, and several other newly identified DAPs (DAP17090, DAP16263, DAP10796, DAP17097, DAP16424) also have occasional localization to the median body, basal bodies or axonemes as well as to the ventral disc, primarily in prophase.

### Novel DAPs are stable structural components of the ventral disc

Components of the ventral disc and lateral crest should include both stable, structural elements and dynamic or regulatory elements. Stable structural components of the ventral disc would be expected to exhibit little protein turnover, whereas dynamic components of the disc, such as regulatory components or MT motors, would be expected to turn over at a faster rate. The lack of protein turnover observed in the live imaging of representative DAP::GFP strains using FRAP (Figure 6) implies that the DAPs identified here are likely structural components of the disc, rather than transiently associated or regulatory elements. This may also indicate that the repair of the ventral disc structure is minimal during interphase.

In contrast, dynamic DAPs may be only loosely associated with the ventral disc structure and could have either regulatory or cell cycle-specific functions. Loosely or transiently associated proteins like these could have been lost in our disc preparation. This might explain why we did not identify several annexins and protein kinases or phosphatases reported to localize to the ventral disc [28], [34], [36], [49]. Other regulatory or dynamic disc-associated proteins may have similar transient associations with the ventral disc characterized by rapid turnover, leading to them to elude identification by the methods employed here. For example, we have recently shown that alpha-2 annexin is only transiently associated with the ventral disc, as it recovers after photobleaching [12].

Although we did not observe protein turnover of disc-localizing DAPs, we did observe some turnover of DAPs when they also localized to non-disc structures. For example, when DAP13981 or DAP17090 localized to the basal bodies or axonemes, fluorescence recovered within several minutes (Figure 6). This localization was only observed in a small proportion of cells, and thus may be cell cycle specific. Thus there are, in some cases, two or more cellular pools of the same DAP – a stable ventral disc-associated pool, and non-disc-associated pool, which is dynamic or transitory. We suggest that DAPs could accumulate at the axonemes prior to mitosis and cell division, and then move to the new dorsal discs when they assemble. Alternatively, dynamic DAPs could regulate the disassembly of the parental disc during cell division and encystation, or contribute to either the generation or maintenance of dynamic conformations of the ventral disc during attachment.

### The lateral crest is comprised of ankyrin repeat and Nek kinase domain-containing proteins

The “lateral crest” is a repetitive structure surrounding the ventral disc that is comprised of a network of fibers of unknown composition and is putatively contractile [10], [21]. As trophozoites skim along a substrate, the ventral disc maintains a domed conformation (visualized live in three dimensions with the beta-giardin::GFP strain in Figure 2). We have recently shown by TIRFM that the lateral crest contacts the surface, forming a critical seal when trophozoites attach [12]. This seal presumably enables or maintains a negative pressure differential underneath the disc.

The novel DAPs (seven ankyrin repeat proteins, two Nek pseudokinases and one novel protein) that localize to the disc perimeter (Table 1 and Figure 4) likely comprise the lateral crest structure that surrounds the ventral disc. The Nek kinase DAP13981 was specifically localized to the lateral crest using negative staining with anti-GFP immunogold labeling (Figure 4). One ankyrin repeat protein (DAP103810) also localizes to the inner region of the ventral disc near the “bare area”. Some of the lateral crest associated DAPs could structurally link the ventral disc to the lateral crest or marginal plate. DAP16424 is one such protein, as it has a regularly spaced, punctate localization around the disc edge, and localizes to the cytoplasmic regions of the anterior axonemes in the presumptive “marginal plate” region. Several lateral crest-associated DAPs also localize with some frequency to other microtubule-based structures. In addition to localizing to the disc perimeter, DAP17097 also localizes occasionally to the median body, suggesting it may have some MT binding capability. DAP17096, DAP17097, and DAP16424 also localize to the basal bodies as well as the lateral crest, although this localization recovers following photobleaching (Figure 6), suggesting that it may be transient.

It is unlikely that the lateral crest has actin-mediated contractile properties. Contrary to prior reports, we observed neither actin nor actin-binding proteins in our proteomic analysis. Further, the lateral crest associated DAPs we identified (Figure 4) have no homology to known actin binding proteins, and “grasping” or “cinching” dynamics of the lateral crest, as evidenced by a contraction of the lateral crest in the X-Y axis, do not occur during *in vitro* attachment [12]. Actin has been reported to localize to the lateral crest and periphery of the disc using heterologous (anti-chicken) antibodies [21], yet such actin antibodies have produced contradictory localization results in *Giardia* – likely due to the divergence of the giardial actin gene [7], [38]. Other actin-associated genes such as myosin or vinculin were also reported to localize to the lateral crest using heterologous antibodies, but homologs are not present in any of the *Giardia* genomes [38], [50], [51]. The recent investigation of actin in *Giardia* using *Giardia*-specific actin antibodies [69] has shown that actin and other actin-related proteins do not localize to the ventral disc or lateral crest.

### Functions of disc-associated proteins in attachment dynamics and disc biogenesis

We are still in the very preliminary stages of understanding the molecular mechanism of how *Giardia* attaches to surfaces, primarily due to a limited comprehension of disc structure, composition, and attachment dynamics. While we have defined novel structural components of the ventral disc, it is unclear whether there are any variations in the conformational dynamics of the primary structural elements of the ventral disc (e.g., microribbons or crossbridges) during attachment. We have shown, nonetheless, that modern protein-tagging approaches [70], [71] are possible with disc-associated proteins, enabling three dimensional live imaging of *in vivo* attachment dynamics using cytological markers of specific structural elements of the ventral disc and lateral crest (Figure 2). Analysis of ventral disc and lateral crest mutants and investigation of the dynamics of disc-associated proteins using live imaging will be central toward testing attachment hypotheses.

The completed *Giardia* genome, combined with new reverse genetic tools to generate dominant negative [72], antisense [73], and morpholino-based knockdowns [74], permits the functional analysis of DAPs in the context of ventral disc-mediated attachment and biogenesis. Specifically, the current inventory of ventral disc components enables investigations into the order of assembly of the primary structural elements of the ventral disc during dorsal disc biogenesis. One would expect that knockdowns of specific components of the ventral disc required for proper disc assembly would result in improperly formed and/or non-functional ventral discs. Ultimately, a more comprehensive understanding of ventral disc biogenesis and the active or passive contribution of the disc to attachment dynamics is of fundamental importance toward developing new classes of anti-giardial compounds.

## Supporting Information

Figure S1 **Multiple sequence alignment of disc-associated Nek kinase homologs.** Nek kinases identified in the ventral disc proteome were aligned to Nek kinases in other representative eukaryotes using MUSCLE [75] and presented using JalView [76].
(PDF)

Figure S2 **Multiple sequence alignment of DIP13 homologs.** The *Giardia* DIP13 homolog was aligned to DIP13 homologs in other representative eukaryotes using MUSCLE [75]. Alignment is presented using JalView [76].
(PDF)

Figure S3 **Beta-, gamma-, and delta-giardin localize to the microribbons.** Negative staining using anti-GFP immunogold labeling of beta-giardin::GFP, delta-giardin::GFP and gamma-giardin::GFP strains show the association of these proteins with the ventral disc microribbons. Scale bar = 200 nm.
(TIF)

Table S1 **Gateway and other oligonucleotide PCR primers for disc-associated proteins (DAPs).** List of forward and reverse oligonucleotide PCR primers used in the construction of the DAP::GFP *Giardia* strains.
(PDF)

Table S2 **Proteins identified in the detergent-extracted disc preparation.** Candidate disc-associated proteins identified in the proteomic analysis of the disc preparation are summarized with protein domain information and GFP localization, if tagged. (MB = median body, AFL = anterior flagella; CFL = caudal flagella; VFL = ventral flagella; and PFL = posteriolateral flagella).
(XLSX)

Video S1 **Z-stack of DAP17090::GFP immunostained with anti-beta-giardin.** Fluorescence microscopy 3D stack of DAP17090::GFP (green) with the ventral disc microribbons localized using an anti-beta-giardin antibody (red). Nuclei are stained with DAPI.
(MOV)

Video S2 **Z-stack of DAP16263::GFP immunostained with anti-beta-giardin.** Fluorescence microscopy 3D stack of DAP16263::GFP (green) with microribbons localized using an anti-beta-giardin antibody (red). Nuclei are stained with DAPI.
(MOV)
