# Supplementary material for: Novel Structural Components of the Ventral Disc and Lateral Crest in Giardia intestinalis
Source: PLoS Negl Trop Dis. 2011 Dec 20;5(12):e1442. doi: 10.1371/journal.pntd.0001442 (PMC3243723; doi:10.1371/journal.pntd.0001442)
Supplement: Figure S1 — Multiple sequence alignment of disc-associated Nek kinase homologs. Nek kinases identified in the ventral disc proteome were aligned to Nek kinases in other representative eukaryotes using MUSCLE [75] and presented using JalView [76]. (PDF) [file pntd.0001442.s001.pdf]

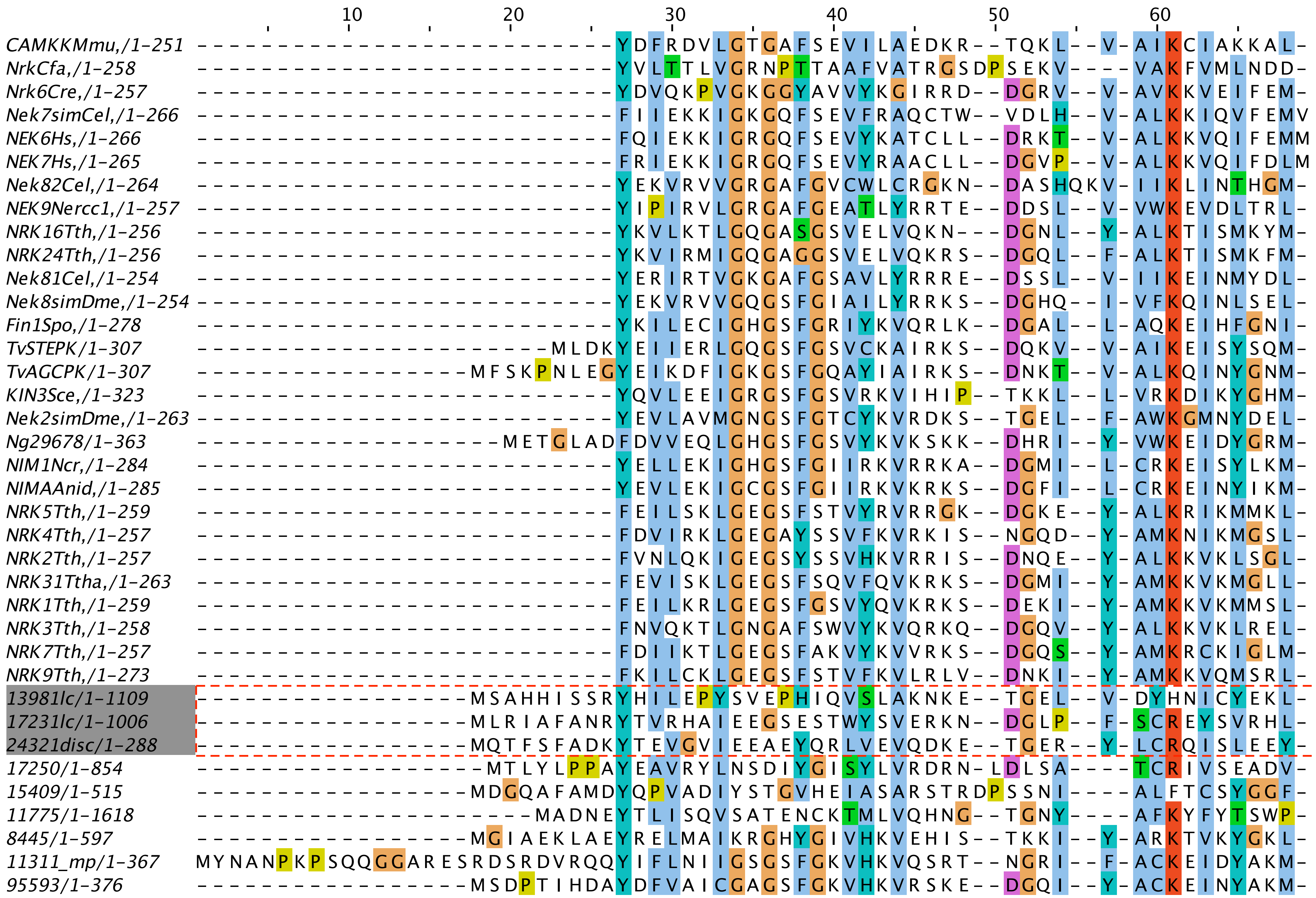

10
20
30
40
50
60
CAMKKMmu,/1-251
NrkCfa,/1-258
Nrk6Cre,/1-257
Nek7simCel,/1-266
NEK6Hs,/1-266
NEK7Hs,/1-265
Nek82Cel,/1-264
NEK9Nercc1,/1-257
NRK16Tth,/1-256
NRK24Tth,/1-256
Nek81Cel,/1-254
Nek8simDme,/1-254
Fin1Spo,/1-278
TvSTEPK/1-307
TvAGCPK/1-307
KIN3Sce,/1-323
Nek2simDme,/1-263
Ng29678/1-363
NIM1Ncr,/1-284
NIMAAnid,/1-285
NRK5Tth,/1-259
NRK4Tth,/1-257
NRK2Tth,/1-257
NRK31Ttha,/1-263
NRK1Tth,/1-259
NRK3Tth,/1-258
NRK7Tth,/1-257
NRK9Tth,/1-273
13981lc/1-1109
17231lc/1-1006
24321disc/1-288
17250/1-854
15409/1-515
11775/1-1618
8445/1-597
11311_mp/1-367
95593/1-376

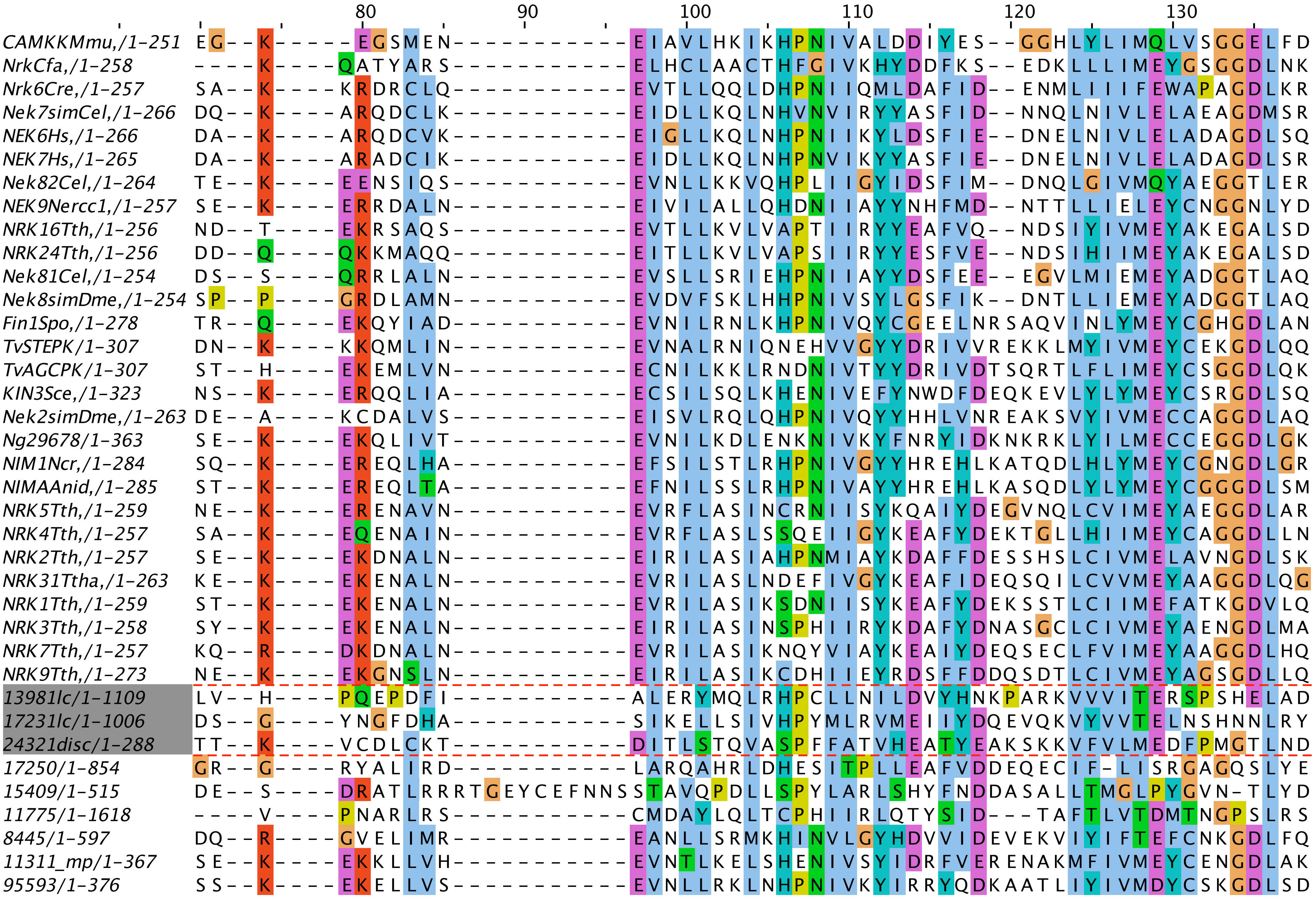
80 90 100 110 120 130
CAMKKMmu,/1-251 EG--K-----EGSMEN-----------EIAVLHKIKHPNIVALDDIYES--GGHLYLIMQLVSGGELFD
NrkCfa,/1-258 ----K----QATYARS-----------ELHCLAACTHFGIVKHYDDFKS--EDKLLLIMEYGSGGDLNK
Nrk6Cre,/1-257 SA--K----KRDRCLQ-----------EVTLLQQLDHPNIIQMLDAFID--ENMLIIIFEWAPAGDLKR
Nek7simCel,/1-266 DQ--K----ARQDCLK-----------EIDLLKQLNHVNVIRYYASFID--NNQLNIVLELAEAGDMSR
NEK6Hs,/1-266 DA--K----ARQDCVK-----------EIGLLKQLNHPNIIKYLDSFIE--DNELNIVLELADAGDLSQ
NEK7Hs,/1-265 DA--K----ARADCIK-----------EIDLLKQLNHPNVIKYYASFIE--DNELNIVLELADAGDLSR
Nek82Cel,/1-264 TE--K----EENSIQS-----------EVNLLKKVQHPLIIGYIDSFIM--DNQLGIVMQYAEGGTLER
NEK9Nercc1,/1-257 SE--K----ERRDALN-----------EIVILALLQHDNIIAYYNHFMD--NTTLLIELEYCNGGNLYD
NRK16Tth,/1-256 ND--T----EKRSAQS-----------EVTLLKVLVAPTIIRYYEAFVQ--NDSIYIVMEYAKEGALSD
NRK24Tth,/1-256 DD--Q----QKKMAQQ-----------EITLLKVLVAPSIIRYYESFVE--NDSIHIIMEYAKEGALSD
Nek81Cel,/1-254 DS--S----QRRLALN-----------EVSLLSRIEHPNIIAYYDSFEE--EGVLMIEMEYADGGTLAQ
Nek8simDme,/1-254 SP--P----GRDLAMN-----------EVDVFSKLHHPNIVSYLGSFIK--DNTLLIEMEYADGGTLAQ
Fin1Spo,/1-278 TR--Q----EKQYIAD-----------EVNILRNLKHPNIVQYCGEELNRSAQVINLYMEYCGHGDLAN
TvSTEPK/1-307 DN--K----KKQMLIN-----------EVNALRNIQNEHVVGYYDRIVVREKKLMYIVMEYCEKGDLQQ
TvAGCPK/1-307 ST--H----EKEMLVN-----------ECNILKKLRNDNIVTYYDRIVDTSQRTLFLIMEYCSGGDLQK
KIN3Sce,/1-323 NS--K----ERQQLIA-----------ECSILSQLKHENIVEFYNWDFDEQKEVLYLYMEYCSRGDLSQ
Nek2simDme,/1-263 DE--A----KCDALVS-----------EISVLRQLQHPNIVQYYHHLVNREAKSVYIVMECCAGGDLAQ
Ng29678/1-363 SE--K----EKQLIVT-----------EVNILKDLENKNIVKYFNRYIDKNKRKLYILMECCEGGDLGK
NIM1Ncr,/1-284 SQ--K----EREQLHA-----------EFSILSTLRHPNIVGYYHREHLKATQDLHLYMEYCGNGDLGR
NIMAAnid,/1-285 ST--K----EREQLTA-----------EFNILSSLRHPNIVAYYHREHLKASQDLYLYMEYCGGGDLSM
NRK5Tth,/1-259 NE--K----ERENAVN-----------EVRFLASINCRNIISYKQAIYDEGVNQLCVIMEYAEGGDLAR
NRK4Tth,/1-257 SA--K----EQENAIN-----------EVRFLASLSSQEIIGYKEAFYDEKTGLLHIIMEYCAGGDLLN
NRK2Tth,/1-257 SE--K----EKDNALN-----------EIRILASIAHPNMIAYKDAFFDESSHSLCIVMELAVNGDLSK
NRK31Ttha,/1-263 KE--K----EKENALN-----------EVRILASLNDEFIVGYKEAFIDEQSQILCVVMEYAAGGDLQG
NRK1Tth,/1-259 ST--K----EKENALN-----------EVRILASIKSDNIISYKEAFYDEKSSTLCIIMEFATKGDVLQ
NRK3Tth,/1-258 SY--K----EKENALN-----------EIRILASINSPHIIRYKDAFYDNASGCLCIVMEYAENGDLMA
NRK7Tth,/1-257 KQ--R----DKDNALN-----------EVRILASIKNQYVIAYKEAIYDEQSECLFVIMEYAAGGDLHQ
NRK9Tth,/1-273 NE--K----EKGNSLN-----------EIRILASIKCDHIIEYRDSFFDDQSDTLCIVMEYAGSGDLLQ
13981lc/1-1109 LV--H----PQEPDFI-----------ALERYMQLRHPCLLNILDVYHNKPARKVVVITERSPSHELAD
17231lc/1-1006 DS--G----YNGFDHA-----------SIKELLSIVHPYMLRVMEIIYDQEVQKVYVVTELNSHNNLRY
24321disc/1-288 TT--K----VCDLCKT-----------DITLSTQVASPFFATVHEATYEAKSKKVFVLMEDFPMGTLND
17250/1-854 GR--G----RYALIRD-----------LARQAHRLDHESITPLLEAFVDDEQECIF-LISRGAGQSLYE
15409/1-515 DE--S----DRATLRRRTGEYCEFNNSSTAVQPDLLSPYLARLSHYFNDDASALLTMGLPYGVN-TLYD
11775/1-1618 ----V----PNARLRS-----------CMDAYLQLTCPHIIRLQTYSID---TAFTLVTDMTNGPSLRS
8445/1-597 DQ--R----GVELIMR-----------EANLLSRMKHINVLGYHDVVIDEVEKVIYIFTEFCNKGDLFQ
11311_mp/1-367 SE--K----EKKLLVH-----------EVNTLKELSHENIVSYIDRFVERENAKMFIVMEYCENGDLAK
95593/1-376 SS--K----EKELLVS-----------EVNLLRKLNHPNIVKYIRRYQDKAATLIYIVMDYCSKGDLSD

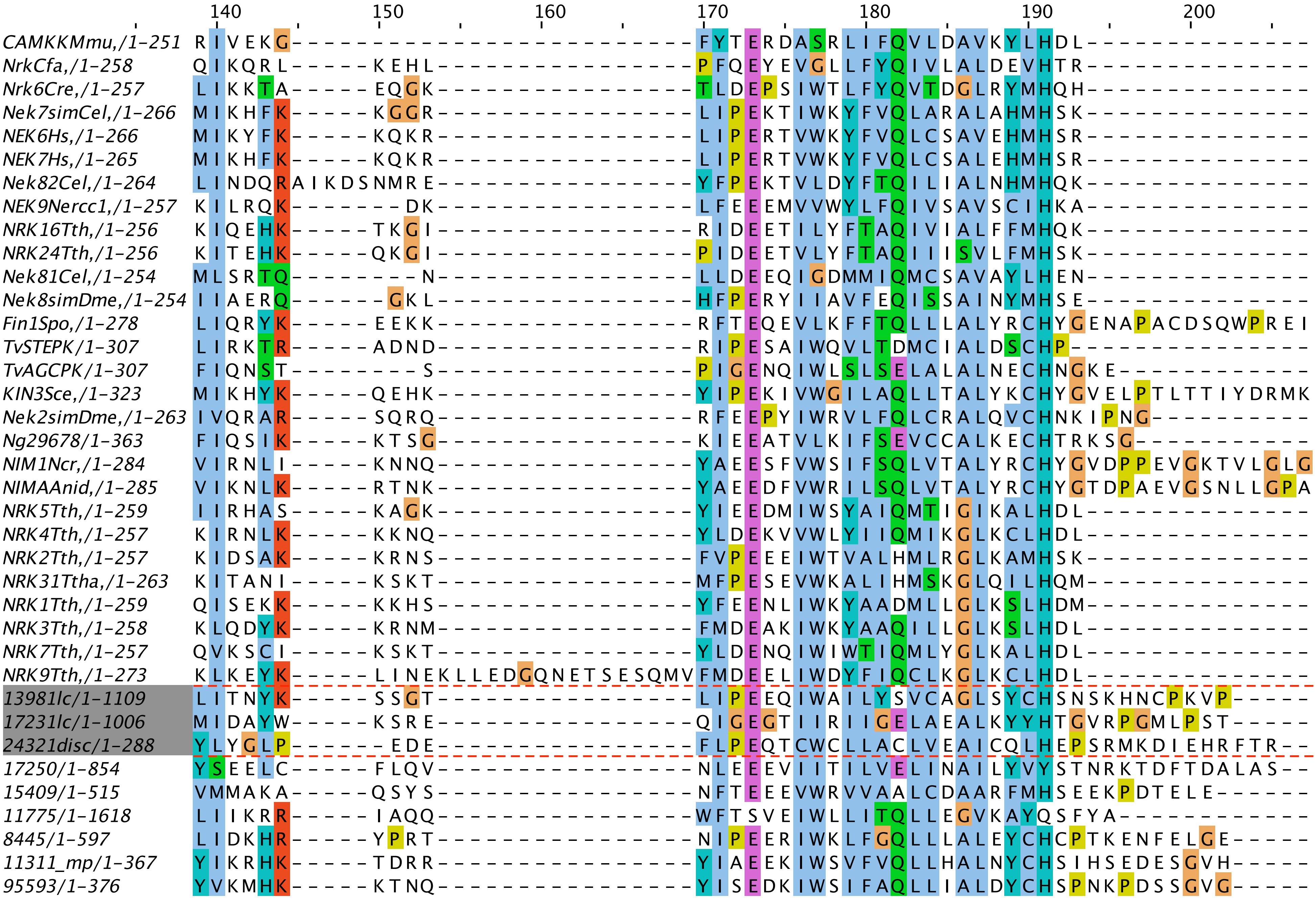
140
150
160
170
180
190
200
CAMKKMmu,/1-251
NrkCfa,/1-258
Nrk6Cre,/1-257
Nek7simCel,/1-266
NEK6Hs,/1-266
NEK7Hs,/1-265
Nek82Cel,/1-264
NEK9Nercc1,/1-257
NRK16Tth,/1-256
NRK24Tth,/1-256
Nek81Cel,/1-254
Nek8simDme,/1-254
Fin1Spo,/1-278
TvSTEPK/1-307
TvAGCPK/1-307
KIN3Sce,/1-323
Nek2simDme,/1-263
Ng29678/1-363
NIM1Ncr,/1-284
NIMAAnid,/1-285
NRK5Tth,/1-259
NRK4Tth,/1-257
NRK2Tth,/1-257
NRK31Ttha,/1-263
NRK1Tth,/1-259
NRK3Tth,/1-258
NRK7Tth,/1-257
NRK9Tth,/1-273
13981lc/1-1109
17231lc/1-1006
24321disc/1-288
17250/1-854
15409/1-515
11775/1-1618
8445/1-597
11311_mp/1-367
95593/1-376

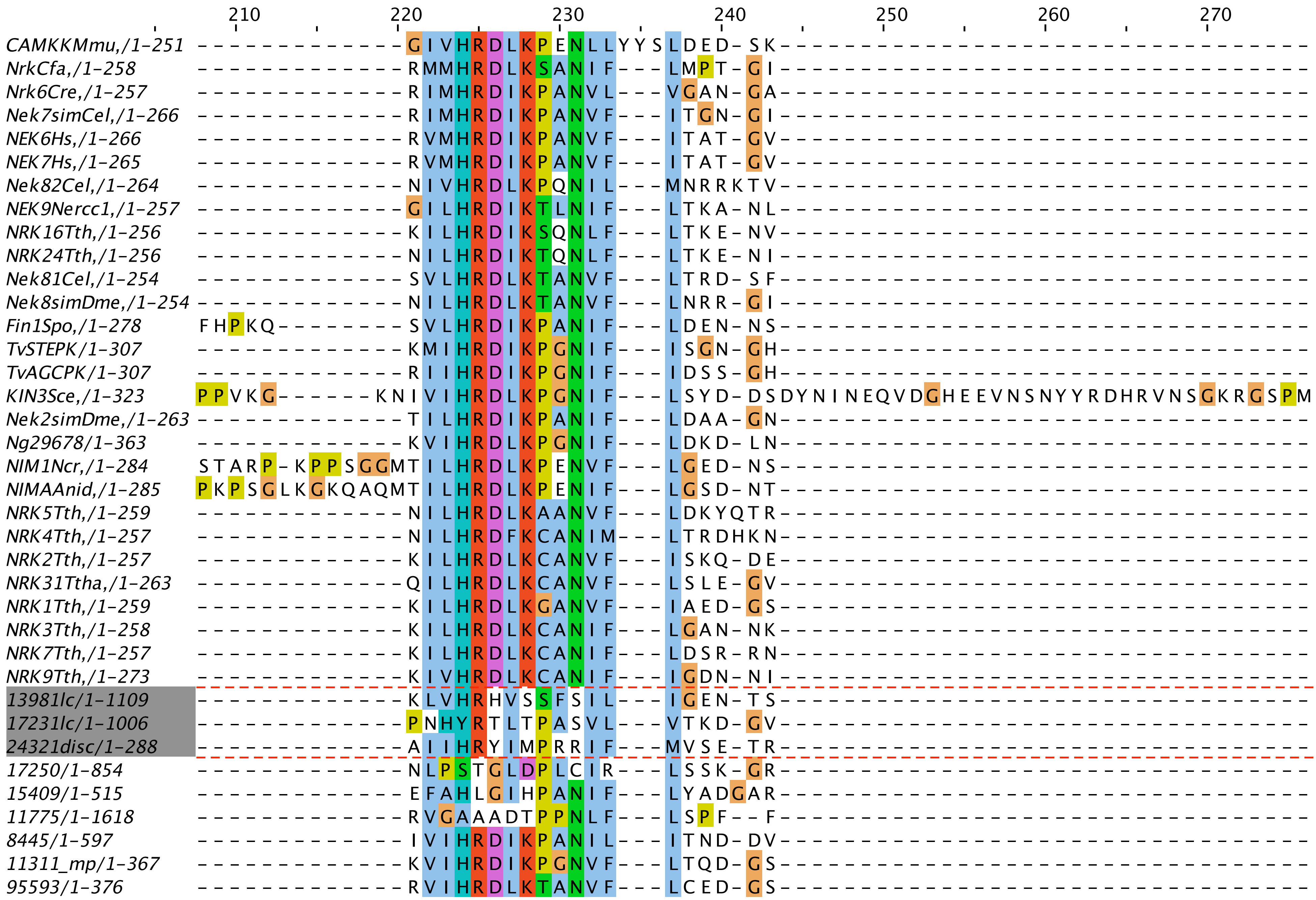

210
220
230
240
250
260
270
CAMKKMmu,/1-251
NrkCfa,/1-258
Nrk6Cre,/1-257
Nek7simCel,/1-266
NEK6Hs,/1-266
NEK7Hs,/1-265
Nek82Cel,/1-264
NEK9Nercc1,/1-257
NRK16Tth,/1-256
NRK24Tth,/1-256
Nek81Cel,/1-254
Nek8simDme,/1-254
Fin1Spo,/1-278
TvSTEPK/1-307
TvAGCPK/1-307
KIN3Sce,/1-323
Nek2simDme,/1-263
Ng29678/1-363
NIM1Ncr,/1-284
NIMAAnid,/1-285
NRK5Tth,/1-259
NRK4Tth,/1-257
NRK2Tth,/1-257
NRK31Ttha,/1-263
NRK1Tth,/1-259
NRK3Tth,/1-258
NRK7Tth,/1-257
NRK9Tth,/1-273
13981lc/1-1109
17231lc/1-1006
24321disc/1-288
17250/1-854
15409/1-515
11775/1-1618
8445/1-597
11311_mp/1-367
95593/1-376

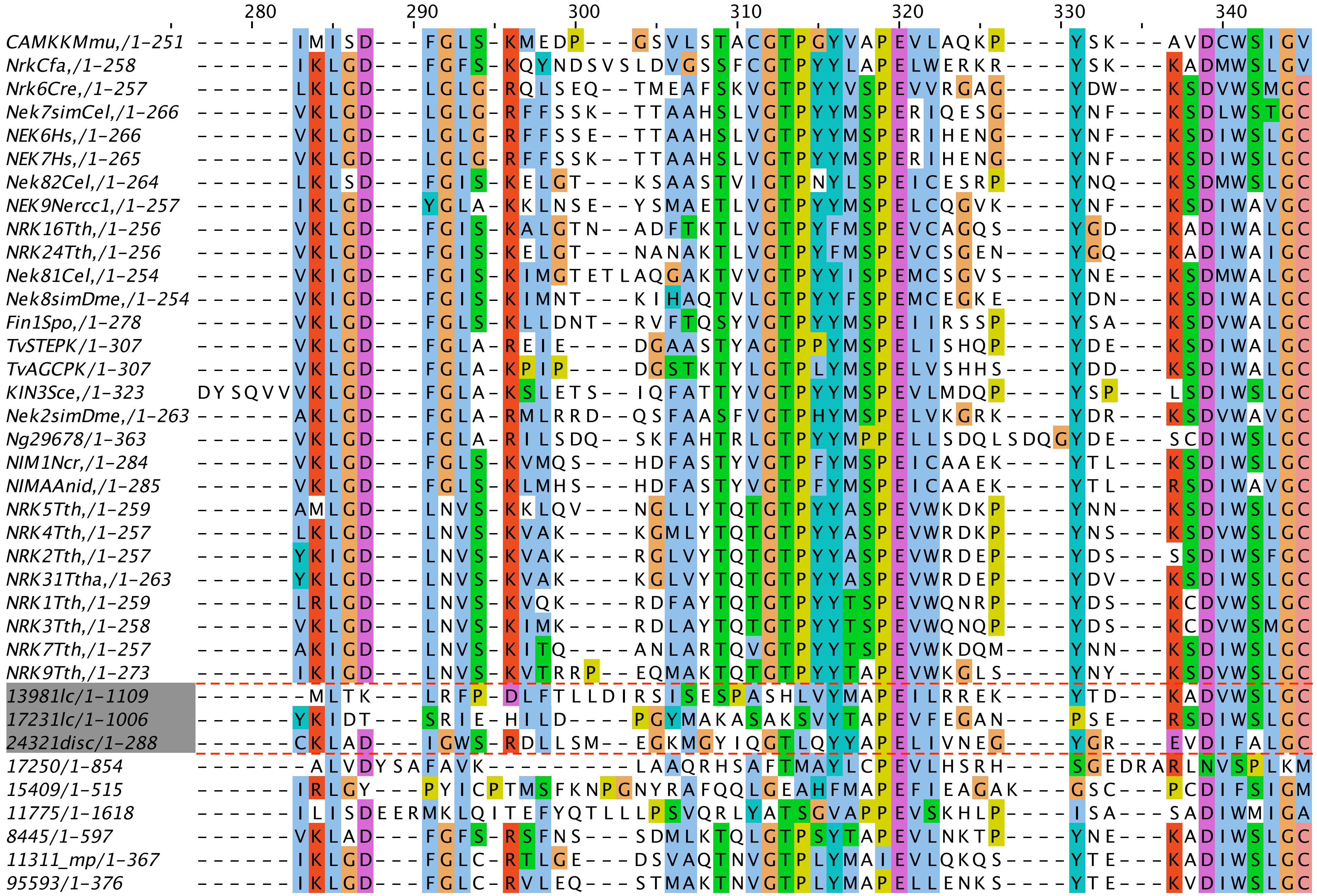

280
290
300
310
320
330
340
CAMKKMmu,/1-251
NrkCfa,/1-258
Nrk6Cre,/1-257
Nek7simCel,/1-266
NEK6Hs,/1-266
NEK7Hs,/1-265
Nek82Cel,/1-264
NEK9Nercc1,/1-257
NRK16Tth,/1-256
NRK24Tth,/1-256
Nek81Cel,/1-254
Nek8simDme,/1-254
Fin1Spo,/1-278
TvSTEPK/1-307
TvAGCPK/1-307
KIN3Sce,/1-323
Nek2simDme,/1-263
Ng29678/1-363
NIM1Ncr,/1-284
NIMAAnid,/1-285
NRK5Tth,/1-259
NRK4Tth,/1-257
NRK2Tth,/1-257
NRK31Ttha,/1-263
NRK1Tth,/1-259
NRK3Tth,/1-258
NRK7Tth,/1-257
NRK9Tth,/1-273
13981lc/1-1109
17231lc/1-1006
24321disc/1-288
17250/1-854
15409/1-515
11775/1-1618
8445/1-597
11311_mp/1-367
95593/1-376

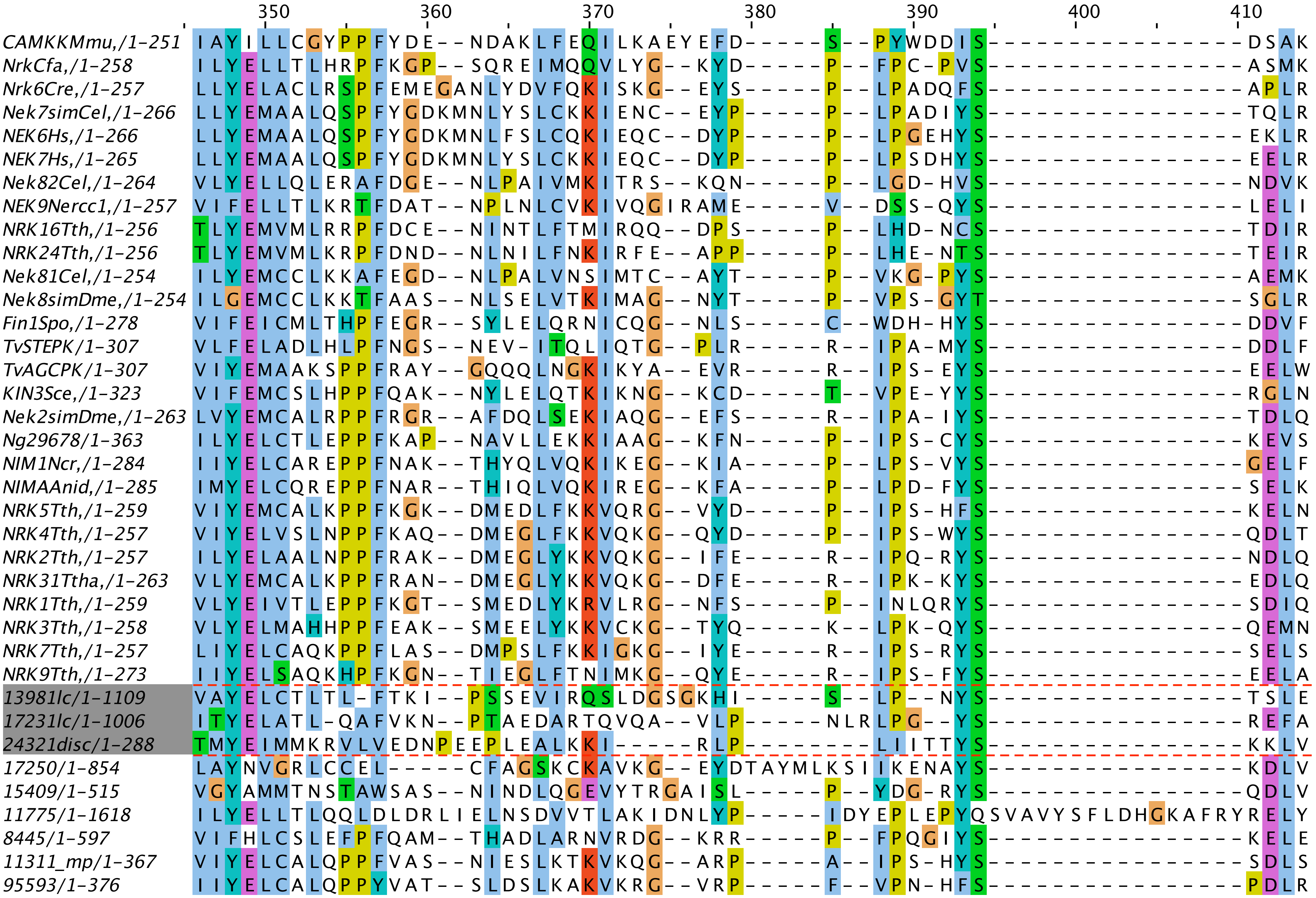
350
360
370
380
390
400
410
CAMKKMmu,/1-251
NrkCfa,/1-258
Nrk6Cre,/1-257
Nek7simCel,/1-266
NEK6Hs,/1-266
NEK7Hs,/1-265
Nek82Cel,/1-264
NEK9Nercc1,/1-257
NRK16Tth,/1-256
NRK24Tth,/1-256
Nek81Cel,/1-254
Nek8simDme,/1-254
Fin1Spo,/1-278
TvSTEPK/1-307
TvAGCPK/1-307
KIN3Sce,/1-323
Nek2simDme,/1-263
Ng29678/1-363
NIM1Ncr,/1-284
NIMAAnid,/1-285
NRK5Tth,/1-259
NRK4Tth,/1-257
NRK2Tth,/1-257
NRK31Ttha,/1-263
NRK1Tth,/1-259
NRK3Tth,/1-258
NRK7Tth,/1-257
NRK9Tth,/1-273
13981lc/1-1109
17231lc/1-1006
24321disc/1-288
17250/1-854
15409/1-515
11775/1-1618
8445/1-597
11311_mp/1-367
95593/1-376

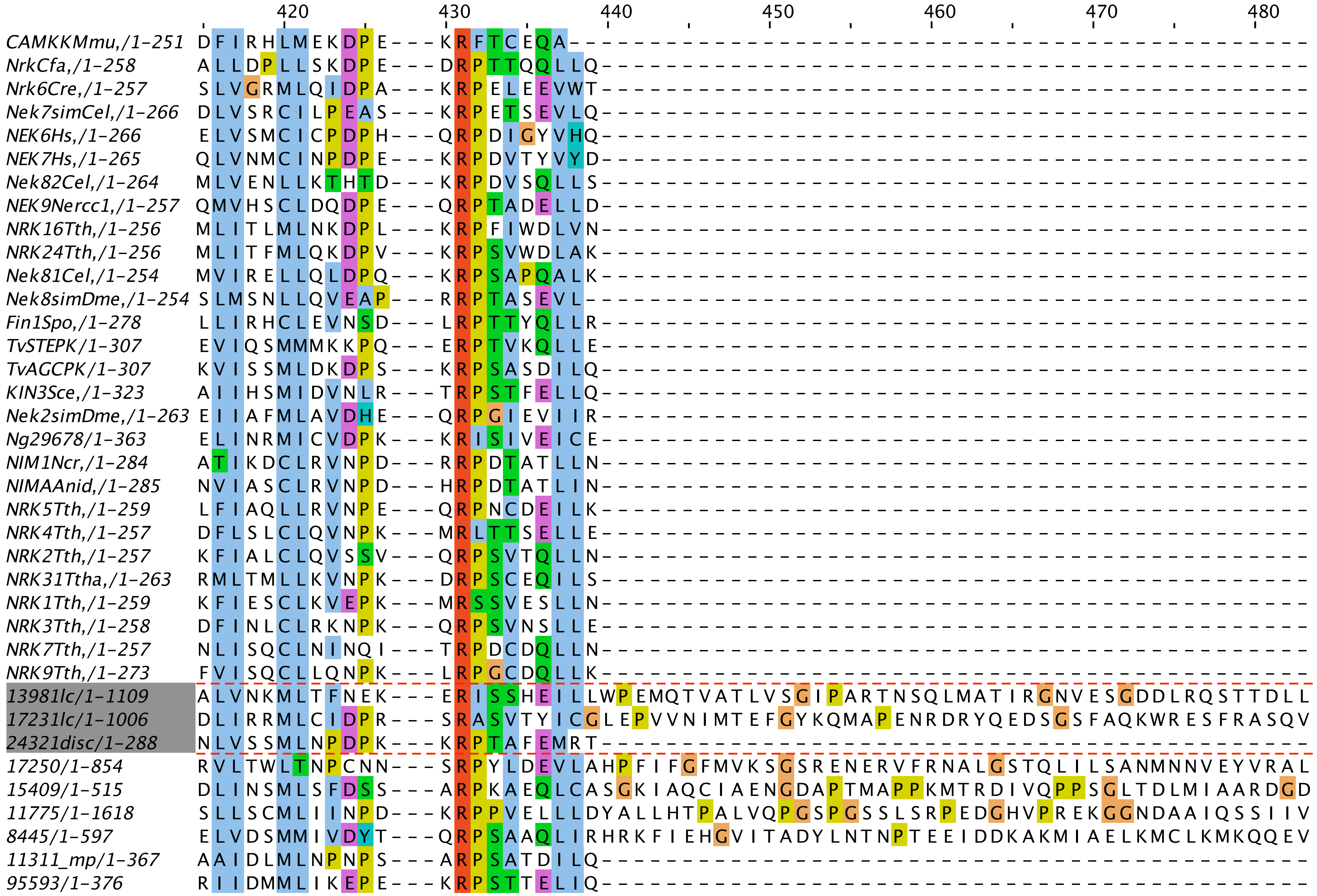
420
430
440
450
460
470
480
CAMKKMmu,/1-251
NrkCfa,/1-258
Nrk6Cre,/1-257
Nek7simCel,/1-266
NEK6Hs,/1-266
NEK7Hs,/1-265
Nek82Cel,/1-264
NEK9Nercc1,/1-257
NRK16Tth,/1-256
NRK24Tth,/1-256
Nek81Cel,/1-254
Nek8simDme,/1-254
Fin1Spo,/1-278
TvSTEPK/1-307
TvAGCPK/1-307
KIN3Sce,/1-323
Nek2simDme,/1-263
Ng29678/1-363
NIM1Ncr,/1-284
NIMAAnid,/1-285
NRK5Tth,/1-259
NRK4Tth,/1-257
NRK2Tth,/1-257
NRK31Ttha,/1-263
NRK1Tth,/1-259
NRK3Tth,/1-258
NRK7Tth,/1-257
NRK9Tth,/1-273
13981lc/1-1109
17231lc/1-1006
24321disc/1-288
17250/1-854
15409/1-515
11775/1-1618
8445/1-597
11311_mp/1-367
95593/1-376

```
                        490       500       510       520       530       540       550
CAMKKMmu,/1-251     ---------------------------------------------------------------------
NrkCfa,/1-258       ---------------------------------------------------------------------
Nrk6Cre,/1-257      ---------------------------------------------------------------------
Nek7simCel,/1-266   ---------------------------------------------------------------------
NEK6Hs,/1-266       ---------------------------------------------------------------------
NEK7Hs,/1-265       ---------------------------------------------------------------------
Nek82Cel,/1-264     ---------------------------------------------------------------------
NEK9Nercc1,/1-257   ---------------------------------------------------------------------
NRK16Tth,/1-256     ---------------------------------------------------------------------
NRK24Tth,/1-256     ---------------------------------------------------------------------
Nek81Cel,/1-254     ---------------------------------------------------------------------
Nek8simDme,/1-254   ---------------------------------------------------------------------
Fin1Spo,/1-278      ---------------------------------------------------------------------
TvSTEPK/1-307       ---------------------------------------------------------------------
TvAGCPK/1-307       ---------------------------------------------------------------------
KIN3Sce,/1-323      ---------------------------------------------------------------------
Nek2simDme,/1-263   ---------------------------------------------------------------------
Ng29678/1-363       ---------------------------------------------------------------------
NIM1Ncr,/1-284      ---------------------------------------------------------------------
NIMAAnid,/1-285     ---------------------------------------------------------------------
NRK5Tth,/1-259      ---------------------------------------------------------------------
NRK4Tth,/1-257      ---------------------------------------------------------------------
NRK2Tth,/1-257      ---------------------------------------------------------------------
NRK31Ttha,/1-263    ---------------------------------------------------------------------
NRK1Tth,/1-259      ---------------------------------------------------------------------
NRK3Tth,/1-258      ---------------------------------------------------------------------
NRK7Tth,/1-257      ---------------------------------------------------------------------
NRK9Tth,/1-273      ---------------------------------------------------------------------
13981lc/1-1109      NRLNATYGKTSATQSSRQTMSKEIQDARD----------------------------------------
17231lc/1-1006      G--------------------------------------------------------------------
24321disc/1-288     ---------------------------------------------------------------------
17250/1-854         V--------------------------------------------------------------------
15409/1-515         L--------------------------------------------------------------------
11775/1-1618        GSDAPDATLQNTLLVGVKTELHKAVEENNLDKLFMNLQFARSTDIDGNTALVYAIKANNEDAVKILAQE
8445/1-597          D--------------------------------------------------------------------
11311_mp/1-367      ---------------------------------------------------------------------
95593/1-376         ---------------------------------------------------------------------
```

```
                      560       570       580       590       600       610
CAMKKMmu,/1-251     ---------------------------------------------------------------------
NrkCfa,/1-258       ---------------------------------------------------------------------
Nrk6Cre,/1-257      ---------------------------------------------------------------------
Nek7simCel,/1-266   ---------------------------------------------------------------------
NEK6Hs,/1-266       ---------------------------------------------------------------------
NEK7Hs,/1-265       ---------------------------------------------------------------------
Nek82Cel,/1-264     ---------------------------------------------------------------------
NEK9Nercc1,/1-257   ---------------------------------------------------------------------
NRK16Tth,/1-256     ---------------------------------------------------------------------
NRK24Tth,/1-256     ---------------------------------------------------------------------
Nek81Cel,/1-254     ---------------------------------------------------------------------
Nek8simDme,/1-254   ---------------------------------------------------------------------
Fin1Spo,/1-278      ---------------------------------------------------------------------
TvSTEPK/1-307       ---------------------------------------------------------------------
TvAGCPK/1-307       ---------------------------------------------------------------------
KIN3Sce,/1-323      ---------------------------------------------------------------------
Nek2simDme,/1-263   ---------------------------------------------------------------------
Ng29678/1-363       ---------------------------------------------------------------------
NIM1Ncr,/1-284      ---------------------------------------------------------------------
NIMAAnid,/1-285     ---------------------------------------------------------------------
NRK5Tth,/1-259      ---------------------------------------------------------------------
NRK4Tth,/1-257      ---------------------------------------------------------------------
NRK2Tth,/1-257      ---------------------------------------------------------------------
NRK31Ttha,/1-263    ---------------------------------------------------------------------
NRK1Tth,/1-259      ---------------------------------------------------------------------
NRK3Tth,/1-258      ---------------------------------------------------------------------
NRK7Tth,/1-257      ---------------------------------------------------------------------
NRK9Tth,/1-273      ---------------------------------------------------------------------
13981lc/1-1109      ---------------------------------------------------------------------
17231lc/1-1006      ---------------------------------------------------------------------
24321disc/1-288     ---------------------------------------------------------------------
17250/1-854         ---------------------------------------------------------------------
15409/1-515         ---------------------------------------------------------------------
11775/1-1618        EIGVLTGEGYSPLHLALLLNHIQLAKYLDASYDLQFLHGVELWVDEHGVSNLMRCVCERNLVGVYYYSD
8445/1-597          ---------------------------------------------------------------------
11311_mp/1-367      ---------------------------------------------------------------------
95593/1-376         ---------------------------------------------------------------------
```

```
                       630       640       650       660       670       680
CAMKKMmu,/1-251   ---------------------------------------------------------------------
NrkCfa,/1-258     ---------------------------------------------------------------------
Nrk6Cre,/1-257    ---------------------------------------------------------------------
Nek7simCel,/1-266 ---------------------------------------------------------------------
NEK6Hs,/1-266     ---------------------------------------------------------------------
NEK7Hs,/1-265     ---------------------------------------------------------------------
Nek82Cel,/1-264   ---------------------------------------------------------------------
NEK9Nercc1,/1-257 ---------------------------------------------------------------------
NRK16Tth,/1-256   ---------------------------------------------------------------------
NRK24Tth,/1-256   ---------------------------------------------------------------------
Nek81Cel,/1-254   ---------------------------------------------------------------------
Nek8simDme,/1-254 ---------------------------------------------------------------------
Fin1Spo,/1-278    ---------------------------------------------------------------------
TvSTEPK/1-307     ---------------------------------------------------------------------
TvAGCPK/1-307     ---------------------------------------------------------------------
KIN3Sce,/1-323    ---------------------------------------------------------------------
Nek2simDme,/1-263 ---------------------------------------------------------------------
Ng29678/1-363     ---------------------------------------------------------------------
NIM1Ncr,/1-284    ---------------------------------------------------------------------
NIMAAnid,/1-285   ---------------------------------------------------------------------
NRK5Tth,/1-259    ---------------------------------------------------------------------
NRK4Tth,/1-257    ---------------------------------------------------------------------
NRK2Tth,/1-257    ---------------------------------------------------------------------
NRK31Ttha,/1-263  ---------------------------------------------------------------------
NRK1Tth,/1-259    ---------------------------------------------------------------------
NRK3Tth,/1-258    ---------------------------------------------------------------------
NRK7Tth,/1-257    ---------------------------------------------------------------------
NRK9Tth,/1-273    ---------------------------------------------------------------------
13981lc/1-1109    ---------------------------------------------------------------------
17231lc/1-1006    ---------------------------------------------------------------------
24321disc/1-288   ---------------------------------------------------------------------
17250/1-854       ---------------------------------------------------------------------
15409/1-515       ---------------------------------------------------------------------
11775/1-1618      KQQKLQDYDGKTALIKAVLNYNEGLPAGQEDVNLAIIRLLRLHEARIQDKRGRTALMYAAECGYLAAID
8445/1-597        ---------------------------------------------------------------------
11311_mp/1-367    ---------------------------------------------------------------------
95593/1-376       ---------------------------------------------------------------------
```

```
                          700       710       720       730       740       750
CAMKKMmu,/1-251      ---------------------------------------------------------------------
NrkCfa,/1-258        ---------------------------------------------------------------------
Nrk6Cre,/1-257       ---------------------------------------------------------------------
Nek7simCel,/1-266    ---------------------------------------------------------------------
NEK6Hs,/1-266        ---------------------------------------------------------------------
NEK7Hs,/1-265        ---------------------------------------------------------------------
Nek82Cel,/1-264      ---------------------------------------------------------------------
NEK9Nercc1,/1-257    ---------------------------------------------------------------------
NRK16Tth,/1-256      ---------------------------------------------------------------------
NRK24Tth,/1-256      ---------------------------------------------------------------------
Nek81Cel,/1-254      ---------------------------------------------------------------------
Nek8simDme,/1-254    ---------------------------------------------------------------------
Fin1Spo,/1-278       ---------------------------------------------------------------------
TvSTEPK/1-307        ---------------------------------------------------------------------
TvAGCPK/1-307        ---------------------------------------------------------------------
KIN3Sce,/1-323       ---------------------------------------------------------------------
Nek2simDme,/1-263    ---------------------------------------------------------------------
Ng29678/1-363        ---------------------------------------------------------------------
NIM1Ncr,/1-284       ---------------------------------------------------------------------
NIMAAnid,/1-285      ---------------------------------------------------------------------
NRK5Tth,/1-259       ---------------------------------------------------------------------
NRK4Tth,/1-257       ---------------------------------------------------------------------
NRK2Tth,/1-257       ---------------------------------------------------------------------
NRK31Ttha,/1-263     ---------------------------------------------------------------------
NRK1Tth,/1-259       ---------------------------------------------------------------------
NRK3Tth,/1-258       ---------------------------------------------------------------------
NRK7Tth,/1-257       ---------------------------------------------------------------------
NRK9Tth,/1-273       ---------------------------------------------------------------------
13981lc/1-1109       ---------------------------------------------------------------------
17231lc/1-1006       ---------------------------------------------------------------------
24321disc/1-288      ---------------------------------------------------------------------
17250/1-854          ---------------------------------------------------------------------
15409/1-515          ---------------------------------------------------------------------
11775/1-1618         VLSGSEAGMRDNEGHNSLEMAFSNRKLEAARRMLLLEPVPHSCPDRQGLTDLMKACIEEDAFSLFCHMT
8445/1-597           ---------------------------------------------------------------------
11311_mp/1-367       ---------------------------------------------------------------------
95593/1-376          ---------------------------------------------------------------------
```

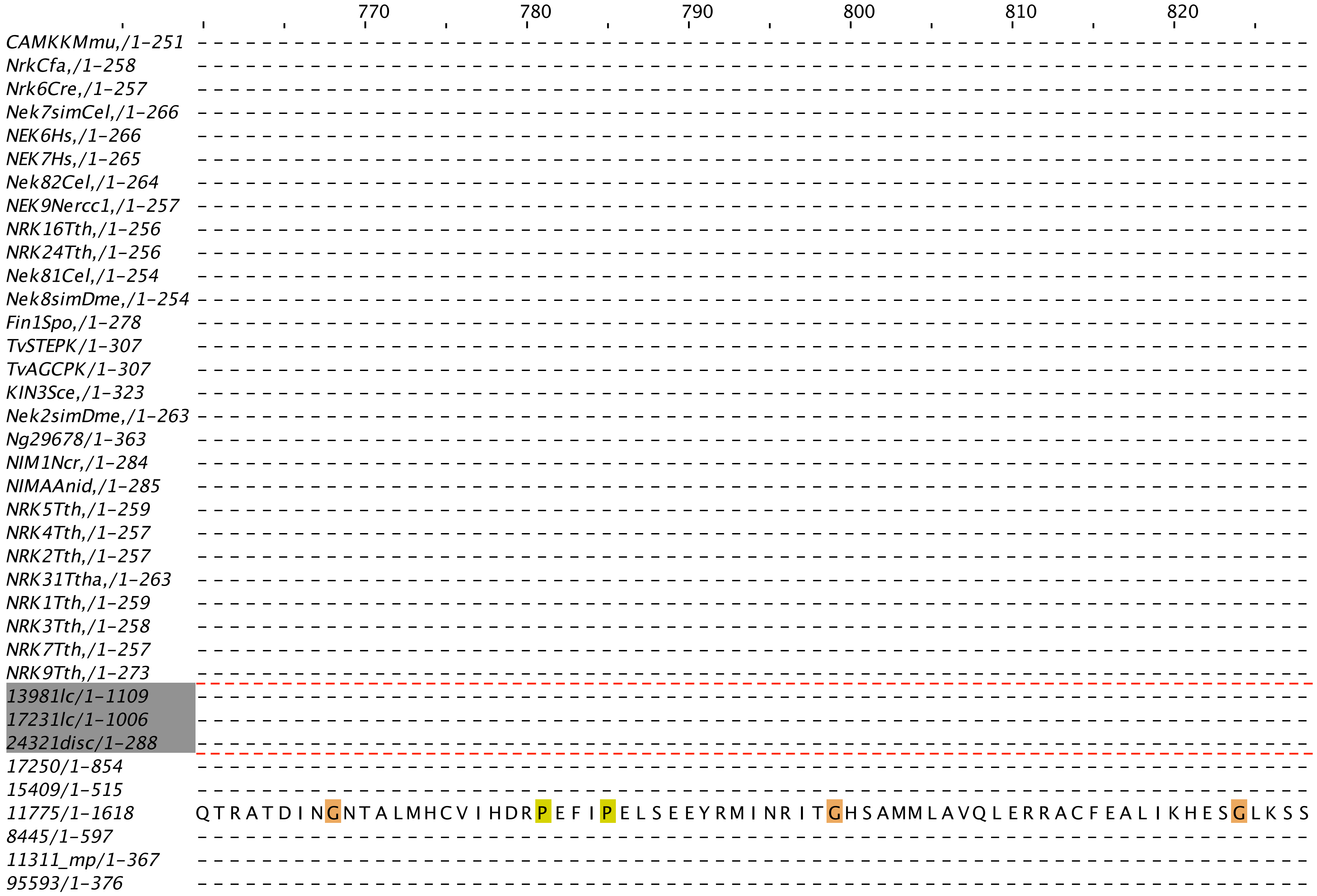

770
780
790
800
810
820
CAMKKMmu,/1-251
NrkCfa,/1-258
Nrk6Cre,/1-257
Nek7simCel,/1-266
NEK6Hs,/1-266
NEK7Hs,/1-265
Nek82Cel,/1-264
NEK9Nercc1,/1-257
NRK16Tth,/1-256
NRK24Tth,/1-256
Nek81Cel,/1-254
Nek8simDme,/1-254
Fin1Spo,/1-278
TvSTEPK/1-307
TvAGCPK/1-307
KIN3Sce,/1-323
Nek2simDme,/1-263
Ng29678/1-363
NIM1Ncr,/1-284
NIMAAnid,/1-285
NRK5Tth,/1-259
NRK4Tth,/1-257
NRK2Tth,/1-257
NRK31Ttha,/1-263
NRK1Tth,/1-259
NRK3Tth,/1-258
NRK7Tth,/1-257
NRK9Tth,/1-273
13981lc/1-1109
17231lc/1-1006
24321disc/1-288
17250/1-854
15409/1-515
11775/1-1618 QTRATDINGNTALMHCVIHDRPEFIPELSEEYRMINRITGHSAMMLAVQLERRACFEALIKHESGLKSS
8445/1-597
11311_mp/1-367
95593/1-376

```
                        830       840       850       860       870       880       890
CAMKKMmu,/1-251     ---------------------------------------------------------------------
NrkCfa,/1-258       ---------------------------------------------------------------------
Nrk6Cre,/1-257      ---------------------------------------------------------------------
Nek7simCel,/1-266   ---------------------------------------------------------------------
NEK6Hs,/1-266       ---------------------------------------------------------------------
NEK7Hs,/1-265       ---------------------------------------------------------------------
Nek82Cel,/1-264     ---------------------------------------------------------------------
NEK9Nercc1,/1-257   ---------------------------------------------------------------------
NRK16Tth,/1-256     ---------------------------------------------------------------------
NRK24Tth,/1-256     ---------------------------------------------------------------------
Nek81Cel,/1-254     ---------------------------------------------------------------------
Nek8simDme,/1-254   ---------------------------------------------------------------------
Fin1Spo,/1-278      ---------------------------------------------------------------------
TvSTEPK/1-307       ---------------------------------------------------------------------
TvAGCPK/1-307       ---------------------------------------------------------------------
KIN3Sce,/1-323      ---------------------------------------------------------------------
Nek2simDme,/1-263   ---------------------------------------------------------------------
Ng29678/1-363       ---------------------------------------------------------------------
NIM1Ncr,/1-284      ---------------------------------------------------------------------
NIMAAnid,/1-285     ---------------------------------------------------------------------
NRK5Tth,/1-259      ---------------------------------------------------------------------
NRK4Tth,/1-257      ---------------------------------------------------------------------
NRK2Tth,/1-257      ---------------------------------------------------------------------
NRK31Ttha,/1-263    ---------------------------------------------------------------------
NRK1Tth,/1-259      ---------------------------------------------------------------------
NRK3Tth,/1-258      ---------------------------------------------------------------------
NRK7Tth,/1-257      ---------------------------------------------------------------------
NRK9Tth,/1-273      ---------------------------------------------------------------------
13981lc/1-1109      ---------------------------------------------------------------------
17231lc/1-1006      ---------------------------------------------------------------------
24321disc/1-288     ---------------------------------------------------------------------
17250/1-854         ---------------------------------------------------------------------
15409/1-515         ---------------------------------------------------------------------
11775/1-1618        SGMTALMMCASQGLRPYVNRLLNIETNIFNGTWNAMTYAIAANHHEIIGLLAPLELNLSAVLKDYDALT
8445/1-597          ---------------------------------------------------------------------
11311_mp/1-367      ---------------------------------------------------------------------
95593/1-376         ---------------------------------------------------------------------
```

```
                    900       910       920       930       940       950       960
CAMKKMmu,/1-251     ---------------------------------------------------------------------
NrkCfa,/1-258       ---------------------------------------------------------------------
Nrk6Cre,/1-257      ---------------------------------------------------------------------
Nek7simCel,/1-266   ---------------------------------------------------------------------
NEK6Hs,/1-266       ---------------------------------------------------------------------
NEK7Hs,/1-265       ---------------------------------------------------------------------
Nek82Cel,/1-264     ---------------------------------------------------------------------
NEK9Nercc1,/1-257   ---------------------------------------------------------------------
NRK16Tth,/1-256     ---------------------------------------------------------------------
NRK24Tth,/1-256     ---------------------------------------------------------------------
Nek81Cel,/1-254     ---------------------------------------------------------------------
Nek8simDme,/1-254   ---------------------------------------------------------------------
Fin1Spo,/1-278      ---------------------------------------------------------------------
TvSTEPK/1-307       ---------------------------------------------------------------------
TvAGCPK/1-307       ---------------------------------------------------------------------
KIN3Sce,/1-323      ---------------------------------------------------------------------
Nek2simDme,/1-263   ---------------------------------------------------------------------
Ng29678/1-363       ---------------------------------------------------------------------
NIM1Ncr,/1-284      ---------------------------------------------------------------------
NIMAAnid,/1-285     ---------------------------------------------------------------------
NRK5Tth,/1-259      ---------------------------------------------------------------------
NRK4Tth,/1-257      ---------------------------------------------------------------------
NRK2Tth,/1-257      ---------------------------------------------------------------------
NRK31Ttha,/1-263    ---------------------------------------------------------------------
NRK1Tth,/1-259      ---------------------------------------------------------------------
NRK3Tth,/1-258      ---------------------------------------------------------------------
NRK7Tth,/1-257      ---------------------------------------------------------------------
NRK9Tth,/1-273      ---------------------------------------------------------------------
13981lc/1-1109      ---------------------------------------------------------------------
17231lc/1-1006      ---------------------------------------------------------------------
24321disc/1-288     ---------------------------------------------------------------------
17250/1-854         ---------------------------------------------------------------------
15409/1-515         ---------------------------------------------------------------------
11775/1-1618        DTAAEIQQELEGAASSVRSTISLNSGYTPLMFAAQQANINAVWGLRDKYLGRKDQHGFTALIYATRVKS
8445/1-597          ---------------------------------------------------------------------
11311_mp/1-367      ---------------------------------------------------------------------
95593/1-376         ---------------------------------------------------------------------
```

```
                          970       980       990       1000      1010      1020      1030
CAMKKMmu,/1-251      ---------------------------------------------------------------------
NrkCfa,/1-258        ---------------------------------------------------------------------
Nrk6Cre,/1-257       ---------------------------------------------------------------------
Nek7simCel,/1-266    ---------------------------------------------------------------------
NEK6Hs,/1-266        ---------------------------------------------------------------------
NEK7Hs,/1-265        ---------------------------------------------------------------------
Nek82Cel,/1-264      ---------------------------------------------------------------------
NEK9Nercc1,/1-257    ---------------------------------------------------------------------
NRK16Tth,/1-256      ---------------------------------------------------------------------
NRK24Tth,/1-256      ---------------------------------------------------------------------
Nek81Cel,/1-254      ---------------------------------------------------------------------
Nek8simDme,/1-254    ---------------------------------------------------------------------
Fin1Spo,/1-278       ---------------------------------------------------------------------
TvSTEPK/1-307        ---------------------------------------------------------------------
TvAGCPK/1-307        ---------------------------------------------------------------------
KIN3Sce,/1-323       ---------------------------------------------------------------------
Nek2simDme,/1-263    ---------------------------------------------------------------------
Ng29678/1-363        ---------------------------------------------------------------------
NIM1Ncr,/1-284       ---------------------------------------------------------------------
NIMAAnid,/1-285      ---------------------------------------------------------------------
NRK5Tth,/1-259       ---------------------------------------------------------------------
NRK4Tth,/1-257       ---------------------------------------------------------------------
NRK2Tth,/1-257       ---------------------------------------------------------------------
NRK31Ttha,/1-263     ---------------------------------------------------------------------
NRK1Tth,/1-259       ---------------------------------------------------------------------
NRK3Tth,/1-258       ---------------------------------------------------------------------
NRK7Tth,/1-257       ---------------------------------------------------------------------
NRK9Tth,/1-273       ---------------------------------------------------------------------
13981lc/1-1109       --------RFIGDPTTRPLSSAVGGQEFHHAVPNISWKSALMRSGQLGNSMTASAVNSPGMIPANQGPD
17231lc/1-1006       ---------------------------------------------------------------------
24321disc/1-288      ---------------------------------------------------------------------
17250/1-854          ----------------------------DKQKRLTNYDGATALMAAAVLGNIEVLKILIPHEFGMRMIT
15409/1-515          ---------------------------------------------------------------------
11775/1-1618         LECCRLLVDERDILCNQGLSAWFYAYQNGYHEPLDIIRPTIKYANTEHRLTDLHMLILRNEVPLSSVQP
8445/1-597           ---------------------------------------------------------------------
11311_mp/1-367       ---------------------------------------------------------------------
95593/1-376          ---------------------------------------------------------------------
```

```
                          1040      1050      1060      1070      1080      1090      1100
CAMKKMmu,/1-251     ---------------------------------------------------------------------
NrkCfa,/1-258       ---------------------------------------------------------------------
Nrk6Cre,/1-257      ---------------------------------------------------------------------
Nek7simCel,/1-266   ---------------------------------------------------------------------
NEK6Hs,/1-266       ---------------------------------------------------------------------
NEK7Hs,/1-265       ---------------------------------------------------------------------
Nek82Cel,/1-264     ---------------------------------------------------------------------
NEK9Nercc1,/1-257   ---------------------------------------------------------------------
NRK16Tth,/1-256     ---------------------------------------------------------------------
NRK24Tth,/1-256     ---------------------------------------------------------------------
Nek81Cel,/1-254     ---------------------------------------------------------------------
Nek8simDme,/1-254   ---------------------------------------------------------------------
Fin1Spo,/1-278      ---------------------------------------------------------------------
TvSTEPK/1-307       ---------------------------------------------------------------------
TvAGCPK/1-307       ---------------------------------------------------------------------
KIN3Sce,/1-323      ---------------------------------------------------------------------
Nek2simDme,/1-263   ---------------------------------------------------------------------
Ng29678/1-363       ---------------------------------------------------------------------
NIM1Ncr,/1-284      ---------------------------------------------------------------------
NIMAAnid,/1-285     ---------------------------------------------------------------------
NRK5Tth,/1-259      ---------------------------------------------------------------------
NRK4Tth,/1-257      ---------------------------------------------------------------------
NRK2Tth,/1-257      ---------------------------------------------------------------------
NRK31Ttha,/1-263    ---------------------------------------------------------------------
NRK1Tth,/1-259      ---------------------------------------------------------------------
NRK3Tth,/1-258      ---------------------------------------------------------------------
NRK7Tth,/1-257      ---------------------------------------------------------------------
NRK9Tth,/1-273      ---------------------------------------------------------------------
13981lc/1-1109      PITKSFAGSHGMTGMTASQLNVADYSAIVANAAKMGGDSAVMKDDVATQILQSRRITEPINDRLATQKY
17231lc/1-1006      -------------PTERKFKNAAEDTGTGMNASQMSMRIQQNLDANSADNRERAAYMTDNVGMYSLSVD
24321disc/1-288     ---------------------------------------------------------------------
17250/1-854         GDSLNGATALILAARNGQTEACKLLAPHEAKLKKRDGFTALMEATVRGHSSAADVLMKYEAKSVIQLDD
15409/1-515         --------------------SGVRNNLHQAGSTDQCGRTALMYAAEHGYADCVRAIVSDAMGHGEVRLQ
11775/1-1618        GGTVSSSLTADSTVRSSSAKSSLHHRDPHEGLQSTYTPNRFRRSQVTTSAILCPKELSNTSNMISLESS
8445/1-597          ---------------------------------------------------------------------
11311_mp/1-367      ---------------------------------------------------------------------
95593/1-376         ---------------------------------------------------------------------
```

```
                      1110      1120      1130      1140      1150      1160      1170
CAMKKMmu,/1-251     ---------------------------------------------------------------------
NrkCfa,/1-258       ---------------------------------------------------------------------
Nrk6Cre,/1-257      ---------------------------------------------------------------------
Nek7simCel,/1-266   ---------------------------------------------------------------------
NEK6Hs,/1-266       ---------------------------------------------------------------------
NEK7Hs,/1-265       ---------------------------------------------------------------------
Nek82Cel,/1-264     ---------------------------------------------------------------------
NEK9Nercc1,/1-257   ---------------------------------------------------------------------
NRK16Tth,/1-256     ---------------------------------------------------------------------
NRK24Tth,/1-256     ---------------------------------------------------------------------
Nek81Cel,/1-254     ---------------------------------------------------------------------
Nek8simDme,/1-254   ---------------------------------------------------------------------
Fin1Spo,/1-278      ---------------------------------------------------------------------
TvSTEPK/1-307       ---------------------------------------------------------------------
TvAGCPK/1-307       ---------------------------------------------------------------------
KIN3Sce,/1-323      ---------------------------------------------------------------------
Nek2simDme,/1-263   ---------------------------------------------------------------------
Ng29678/1-363       ---------------------------------------------------------------------
NIM1Ncr,/1-284      ---------------------------------------------------------------------
NIMAAnid,/1-285     ---------------------------------------------------------------------
NRK5Tth,/1-259      ---------------------------------------------------------------------
NRK4Tth,/1-257      ---------------------------------------------------------------------
NRK2Tth,/1-257      ---------------------------------------------------------------------
NRK31Ttha,/1-263    ---------------------------------------------------------------------
NRK1Tth,/1-259      ---------------------------------------------------------------------
NRK3Tth,/1-258      ---------------------------------------------------------------------
NRK7Tth,/1-257      ---------------------------------------------------------------------
NRK9Tth,/1-273      ---------------------------------------------------------------------
13981lc/1-1109      TKK--TTDMEEVAPIVVNEIMSDFLTMRYGVQVDKEHLVDRIFNVVPGKIVNADKADLDPEAPDVQRDI
17231lc/1-1006      DLISSHSNVPGVRNEPVTRSSLTPGAAVATTLYAAPTYSREATRIISSRVDSMRTGAELPGNTSINNGL
24321disc/1-288     ---------------------------------------------------------------------
17250/1-854         LSNPKNGT-------------------------------------------------------------
15409/1-515         DKYMWTAT-------------------------------------------------------------
11775/1-1618        LVLPEDPPTKTQPDYLQDVSTLLYLTKLYTKSGETALMLAAKSYRGKMDKNLLALEAGMRDSYGRLASC
8445/1-597          ---------------------------------------------------------------------
11311_mp/1-367      ---------------------------------------------------------------------
95593/1-376         ---------------------------------------------------------------------
```

```
                        1180      1190      1200      1210      1220      1230      1240
CAMKKMmu,/1-251         ---------------------------------------------------------------------
NrkCfa,/1-258           ---------------------------------------------------------------------
Nrk6Cre,/1-257          ---------------------------------------------------------------------
Nek7simCel,/1-266       ---------------------------------------------------------------------
NEK6Hs,/1-266           ---------------------------------------------------------------------
NEK7Hs,/1-265           ---------------------------------------------------------------------
Nek82Cel,/1-264         ---------------------------------------------------------------------
NEK9Nercc1,/1-257       ---------------------------------------------------------------------
NRK16Tth,/1-256         ---------------------------------------------------------------------
NRK24Tth,/1-256         ---------------------------------------------------------------------
Nek81Cel,/1-254         ---------------------------------------------------------------------
Nek8simDme,/1-254       ---------------------------------------------------------------------
Fin1Spo,/1-278          ---------------------------------------------------------------------
TvSTEPK/1-307           ---------------------------------------------------------------------
TvAGCPK/1-307           ---------------------------------------------------------------------
KIN3Sce,/1-323          ---------------------------------------------------------------------
Nek2simDme,/1-263       ---------------------------------------------------------------------
Ng29678/1-363           ---------------------------------------------------------------------
NIM1Ncr,/1-284          ---------------------------------------------------------------------
NIMAAnid,/1-285         ---------------------------------------------------------------------
NRK5Tth,/1-259          ---------------------------------------------------------------------
NRK4Tth,/1-257          ---------------------------------------------------------------------
NRK2Tth,/1-257          ---------------------------------------------------------------------
NRK31Ttha,/1-263        ---------------------------------------------------------------------
NRK1Tth,/1-259          ---------------------------------------------------------------------
NRK3Tth,/1-258          ---------------------------------------------------------------------
NRK7Tth,/1-257          ---------------------------------------------------------------------
NRK9Tth,/1-273          ---------------------------------------------------------------------
13981lc/1-1109          RRHSPNDIVDLLMMKKELDERLEPYAYRLSNSSESRARDRYKQSREKESYEPHLTGDHTLRTIENDVQS
17231lc/1-1006          SPSFRNLLEMNSASHNYDNSIDDTSPIYNQNDGSVRATNDAQGAADTDITGMYRDELAYRRRQSGGNTS
24321disc/1-288         ---------------------------------------------------------------------
17250/1-854             ---------------------------------------------------------------------
15409/1-515             ---------------------------------------------------------------------
11775/1-1618            IAIERGNTEFLTEVLPYERDALIADGFTDLMLSILEDDYVALMHHLKNPEQQCGQVVMSAYTALNICIY
8445/1-597              ---------------------------------------------------------------------
11311_mp/1-367          ---------------------------------------------------------------------
95593/1-376             ---------------------------------------------------------------------
```

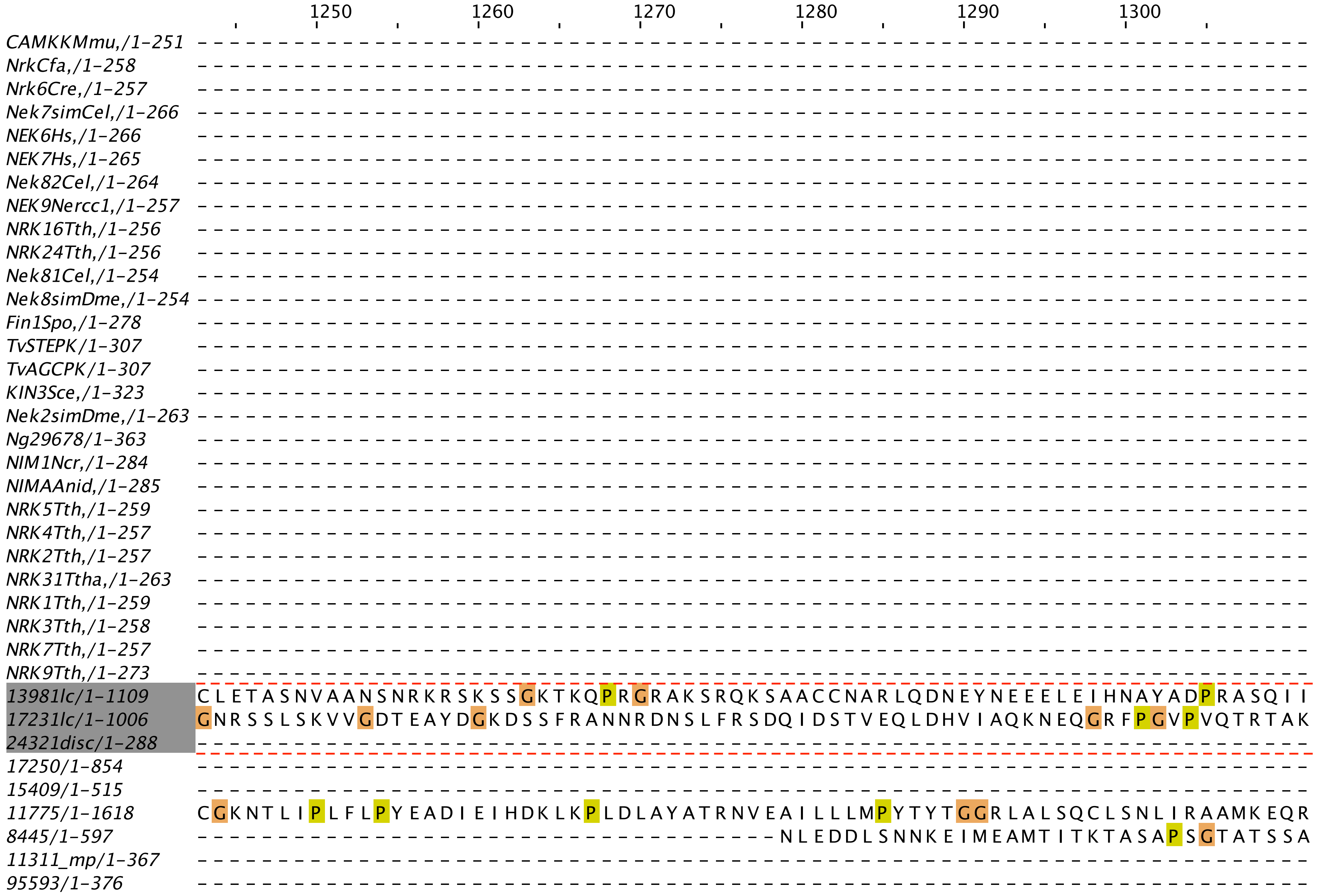
1250
1260
1270
1280
1290
1300
CAMKKMmu,/1-251
NrkCfa,/1-258
Nrk6Cre,/1-257
Nek7simCel,/1-266
NEK6Hs,/1-266
NEK7Hs,/1-265
Nek82Cel,/1-264
NEK9Nercc1,/1-257
NRK16Tth,/1-256
NRK24Tth,/1-256
Nek81Cel,/1-254
Nek8simDme,/1-254
Fin1Spo,/1-278
TvSTEPK/1-307
TvAGCPK/1-307
KIN3Sce,/1-323
Nek2simDme,/1-263
Ng29678/1-363
NIM1Ncr,/1-284
NIMAAnid,/1-285
NRK5Tth,/1-259
NRK4Tth,/1-257
NRK2Tth,/1-257
NRK31Ttha,/1-263
NRK1Tth,/1-259
NRK3Tth,/1-258
NRK7Tth,/1-257
NRK9Tth,/1-273
13981lc/1-1109 CLETASNVAANSNRKRSKSSGKTKQPRGRAKSRQKSAACCNARLQDNEYNEEELEIHNAYADPRASQII
17231lc/1-1006 GNRSSLSKVVGDTEAYDGKDSSFRANNRDNSLFRSDQIDSTVEQLDHVIAQKNEQGRFPGVPVQTRTAK
24321disc/1-288
17250/1-854
15409/1-515
11775/1-1618 CGKNTLIPLFLPYEADIEIHDKLKPLDLAYATRNVEAILLLMPYTYTGGRLALSQCLSNLIRAAMKEQR
8445/1-597 NLEDDLSNNKEIMEAMTITKTASAPSGTATSSA
11311_mp/1-367
95593/1-376

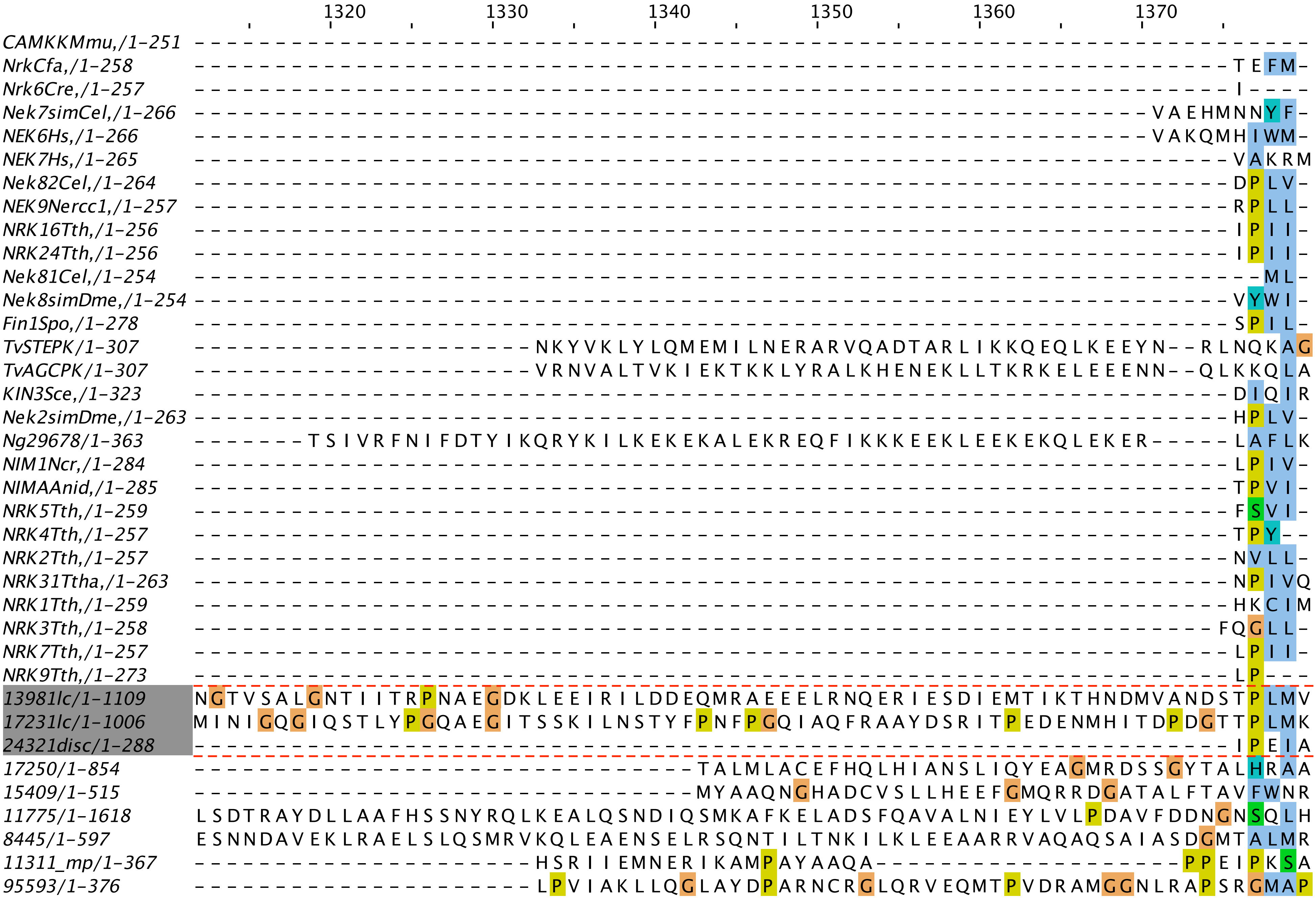
1320 1330 1340 1350 1360 1370
CAMKKMmu,/1-251 ---
NrkCfa,/1-258 --TEFM-
Nrk6Cre,/1-257 --I----
Nek7simCel,/1-266 ---VAEHMNNYF-
NEK6Hs,/1-266 ---VAKQMHIWM-
NEK7Hs,/1-265 --VAKRM
Nek82Cel,/1-264 --DPLV-
NEK9Nercc1,/1-257 --RPLL-
NRK16Tth,/1-256 --IPII-
NRK24Tth,/1-256 --IPII-
Nek81Cel,/1-254 --ML-
Nek8simDme,/1-254 --VYWI-
Fin1Spo,/1-278 --SPIL-
TvSTEPK/1-307 ---------------------NKYVKLYLQMEMILNERARVQADTARLIKKQEQLKEEYN--RLNQKAG
TvAGCPK/1-307 ---------------------VRNVALTVKIEKTKKLYRALKHENEKLLTKRKELEEENN--QLKKQLA
KIN3Sce,/1-323 --DIQIR
Nek2simDme,/1-263 --HPLV-
Ng29678/1-363 -------TSIVRFNIFDTYIKQRYKILKEKEKALEKREQFIKKKEEKLEEKEKQLEKER-----LAFLK
NIM1Ncr,/1-284 --LPIV-
NIMAAnid,/1-285 --TPVI-
NRK5Tth,/1-259 --FSVI-
NRK4Tth,/1-257 --TPY--
NRK2Tth,/1-257 --NVLL-
NRK31Ttha,/1-263 --NPIVQ
NRK1Tth,/1-259 --HKCIM
NRK3Tth,/1-258 ---FQGLL-
NRK7Tth,/1-257 --LPII-
NRK9Tth,/1-273 --LP---
13981lc/1-1109 NGTVSALGNTIITRPNAEGDKLEEIRILDDEQMRAEEELRNQERIESDIEMTIKTHNDMVANDSTPLMV
17231lc/1-1006 MINIGQGIQSTLYPGQAEGITSSKILNSTYFPNFPGQIAQFRAAYDSRITPEDENMHITDPDGTTPLMK
24321disc/1-288 --IPEIA
17250/1-854 -------------------------------TALMLACEFHQLHIANSLIQYEAGMRDSSGYTALHRAA
15409/1-515 -------------------------------MYAAQNGHADCVSLLHEEFGMQRRDGATALFTAVFWNR
11775/1-1618 LSDTRAYDLLAAFHSSNYRQLKEALQSNDIQSMKAFKELADSFQAVALNIEYLVLPDAVFDDNGNSQLH
8445/1-597 ESNNDAVEKLRAELSLQSMRVKQLEAENSELRSQNTILTNKILKLEEAARRVAQAQSAIASDGMTALMR
11311_mp/1-367 ---------------------HSRIIEMNERIKAMPAYAAQA-------------------PPEIPKSA
95593/1-376 ---------------------LPVIAKLLQGLAYDPARNCRGLQRVEQMTPVDRAMGGNLRAPSRGMAP

```
                          1390      1400      1410      1420      1430      1440
CAMKKMmu,/1-251   ---------------------------------------------------------------------
NrkCfa,/1-258     ---------------------------------------------------------------------
Nrk6Cre,/1-257    ---------------------------------------------------------------------
Nek7simCel,/1-266 ---------------------------------------------------------------------
NEK6Hs,/1-266     ---------------------------------------------------------------------
NEK7Hs,/1-265     HAC------------------------------------------------------------------
Nek82Cel,/1-264   ---------------------------------------------------------------------
NEK9Nercc1,/1-257 ---------------------------------------------------------------------
NRK16Tth,/1-256   ---------------------------------------------------------------------
NRK24Tth,/1-256   ---------------------------------------------------------------------
Nek81Cel,/1-254   ---------------------------------------------------------------------
Nek8simDme,/1-254 ---------------------------------------------------------------------
Fin1Spo,/1-278    ---------------------------------------------------------------------
TvSTEPK/1-307     RVFFNE---------------------------------------------------------------
TvAGCPK/1-307     ---------------------------------------------------------------------
KIN3Sce,/1-323    TAR------------------------------------------------------------------
Nek2simDme,/1-263 ---------------------------------------------------------------------
Ng29678/1-363     EKTEFEKQKQTKSVDMTSGLSPLTELNKENIYQTFKI--------------------------------
NIM1Ncr,/1-284    ---------------------------------------------------------------------
NIMAAnid,/1-285   ---------------------------------------------------------------------
NRK5Tth,/1-259    ---------------------------------------------------------------------
NRK4Tth,/1-257    ---------------------------------------------------------------------
NRK2Tth,/1-257    ---------------------------------------------------------------------
NRK31Ttha,/1-263  RNGGE----------------------------------------------------------------
NRK1Tth,/1-259    ---------------------------------------------------------------------
NRK3Tth,/1-258    ---------------------------------------------------------------------
NRK7Tth,/1-257    ---------------------------------------------------------------------
NRK9Tth,/1-273    ---------------------------------------------------------------------
13981lc/1-1109    AASADNVDQVRFLLPMHGGETTTKGITALMMAASAGHVRSVKLLIPKEGRMQDKDGMTALMYAAHFGKL
17231lc/1-1006    AVIRDDMRLVQKYLPTQCRIANDRGVTALMLAAYFDNFDAIQLLLDKESKTQDSAGMTALMYAAHKGNT
24321disc/1-288   NILRGELSA------------------------------------------------------------
17250/1-854       ALGLVSAVKLLITTKECGLRDPKGQTALMFAAQYGHSKCVELLRKEKEAGLVTDKGWSALMSAITNGHT
15409/1-515       CECVKVLAPTEAIISTNDRYWQGERYTAAMEAARWGRPECLQELLGYIDKYTTDNNGNDVAYYAMHPWE
11775/1-1618      IAALAADTRLAAAFVHLHSLRNKKGETALMLAARSGSEAVVDILLNYEAEMQDNNKMTATEHSIVSGTF
8445/1-597        AVQMNDIAGIKAHIATDAGKKNRKGKTALMMCSDTGKTEAAALLIPYEAGTKMSDGTTALILAAIAGHV
11311_mp/1-367    PPGGGHAQ-----RERELREWEERLNTKEQSLRAREEHVDRTLAALNAGTYKGY---------------
95593/1-376       PPSAGYKPVNGRYSDGDLDAWEDRLKEREAALANWEDAISEKETNLKERLAKAGLR-------------
```

```
                      1460      1470      1480      1490      1500      1510
CAMKKMmu,/1-251       ---------------------------------------------------------------------
NrkCfa,/1-258         ---------------------------------------------------------------------
Nrk6Cre,/1-257        ---------------------------------------------------------------------
Nek7simCel,/1-266     ---------------------------------------------------------------------
NEK6Hs,/1-266         ---------------------------------------------------------------------
NEK7Hs,/1-265         ---------------------------------------------------------------------
Nek82Cel,/1-264       ---------------------------------------------------------------------
NEK9Nercc1,/1-257     ---------------------------------------------------------------------
NRK16Tth,/1-256       ---------------------------------------------------------------------
NRK24Tth,/1-256       ---------------------------------------------------------------------
Nek81Cel,/1-254       ---------------------------------------------------------------------
Nek8simDme,/1-254     ---------------------------------------------------------------------
Fin1Spo,/1-278        ---------------------------------------------------------------------
TvSTEPK/1-307         ---------------------------------------------------------------------
TvAGCPK/1-307         ---------------------------------------------------------------------
KIN3Sce,/1-323        ---------------------------------------------------------------------
Nek2simDme,/1-263     ---------------------------------------------------------------------
Ng29678/1-363         ---------------------------------------------------------------------
NIM1Ncr,/1-284        ---------------------------------------------------------------------
NIMAAnid,/1-285       ---------------------------------------------------------------------
NRK5Tth,/1-259        ---------------------------------------------------------------------
NRK4Tth,/1-257        ---------------------------------------------------------------------
NRK2Tth,/1-257        ---------------------------------------------------------------------
NRK31Ttha,/1-263      ---------------------------------------------------------------------
NRK1Tth,/1-259        ---------------------------------------------------------------------
NRK3Tth,/1-258        ---------------------------------------------------------------------
NRK7Tth,/1-257        ---------------------------------------------------------------------
NRK9Tth,/1-273        ---------------------------------------------------------------------
13981lc/1-1109        EAVKELVEAEHNKVNSAGLTALMIAAEFGNVDIVNFLKTYESKKFSASRETAMMRAAKLGHVDIVKELL
17231lc/1-1006        NSVVQLYTHEARMVTGKGVTALMIAAECGWVDIVRTLMPREARLRDDFGNTALIYACKAGRASVVQELL
24321disc/1-288       ---------------------------------------------------------------------
17250/1-854           EAAKLLIDYEGHLTLNMGYTPLMCAVEHKQYEVIDLLKHSQARKTIAAESEEDSWHNGATALMLAAYSN
15409/1-515           HVSADKSSRVREVLNL-----------------------------------------------------
11775/1-1618          HKVLNLYTREKAFLVNECELSPLMLAVLEGDIKTVSAGLKYAKLRTSSGMTALHMAIYLRQQAHANLLK
8445/1-597            DIVNILKKKEGKMQARQGDTALINAAVTGHAEVVRALVEIEAGLRMDDGRAAIHNAAYKGHLECVKILL
11311_mp/1-367        ---------------------------------------------------------------------
95593/1-376           ---------------------------------------------------------------------
```

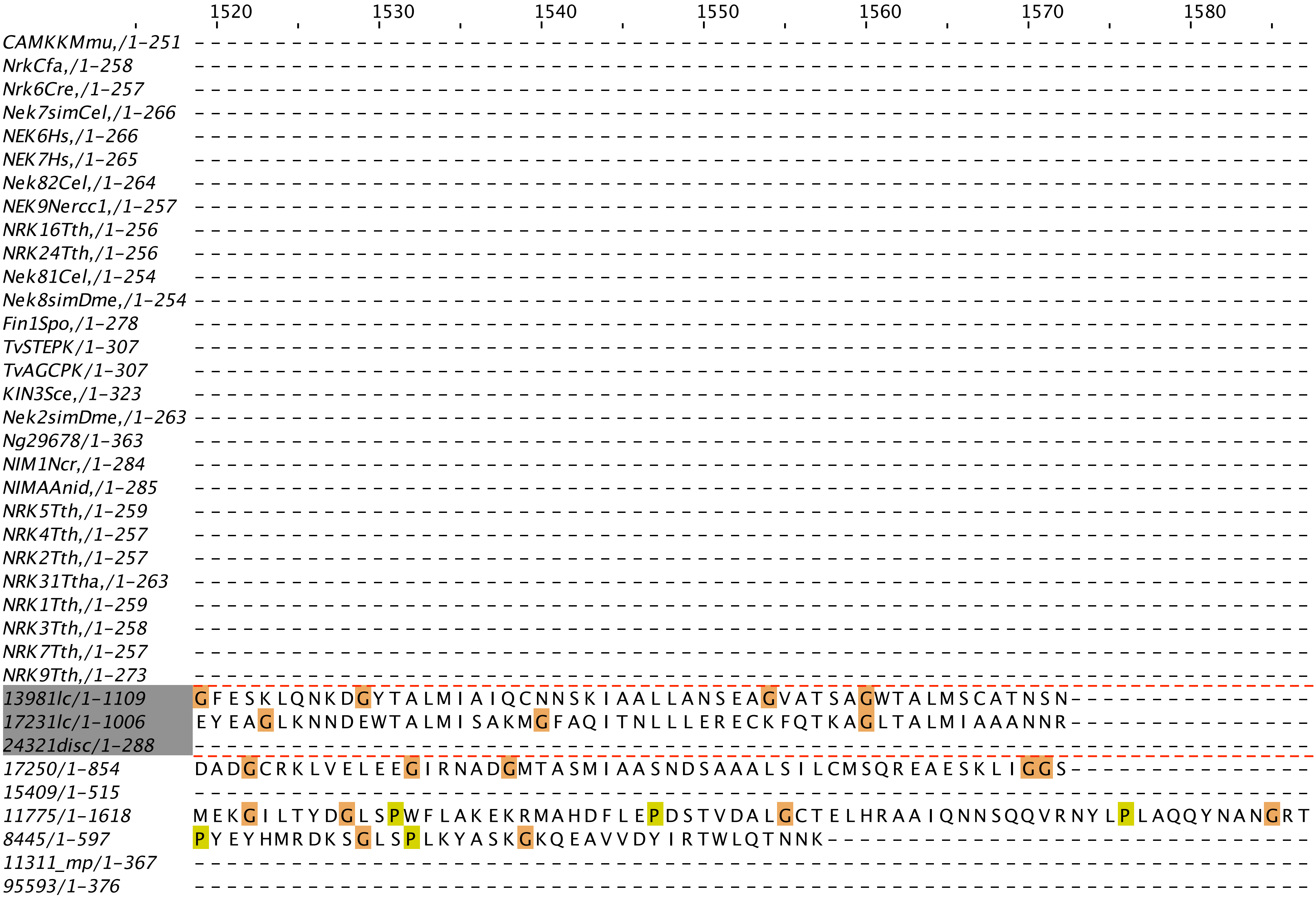
1520 1530 1540 1550 1560 1570 1580
CAMKKMmu,/1-251 --
NrkCfa,/1-258 --
Nrk6Cre,/1-257 --
Nek7simCel,/1-266 --
NEK6Hs,/1-266 --
NEK7Hs,/1-265 --
Nek82Cel,/1-264 --
NEK9Nercc1,/1-257 --
NRK16Tth,/1-256 --
NRK24Tth,/1-256 --
Nek81Cel,/1-254 --
Nek8simDme,/1-254 --
Fin1Spo,/1-278 --
TvSTEPK/1-307 --
TvAGCPK/1-307 --
KIN3Sce,/1-323 --
Nek2simDme,/1-263 --
Ng29678/1-363 --
NIM1Ncr,/1-284 --
NIMAAnid,/1-285 --
NRK5Tth,/1-259 --
NRK4Tth,/1-257 --
NRK2Tth,/1-257 --
NRK31Ttha,/1-263 --
NRK1Tth,/1-259 --
NRK3Tth,/1-258 --
NRK7Tth,/1-257 --
NRK9Tth,/1-273 --
13981lc/1-1109 GFESKLQNKDGYTALMIAIQCNNSKIAALLANSEAGVATSAGWTALMSCATNSN--------------
17231lc/1-1006 EYEAGLKNNDEWTALMISAKMGFAQITNLLLERECKFQTKAGLTALMIAAANNR--------------
24321disc/1-288 --
17250/1-854 DADGCRKLVELEEGIRNADGMTASMIAASNDSAAALSILCMSQREAESKLIGGS--------------
15409/1-515 --
11775/1-1618 MEKGILTYDGLSPWFLAKEKRMAHDFLEPDSTVDALGCTELHRAAIQNNSQQVRNYLPLAQQYNANGRT
8445/1-597 PYEYHMRDKSGLSPLKYASKGKQEAVVDYIRTWLQTNNK-----------------------------
11311_mp/1-367 --
95593/1-376 --

```
                  1590      1600      1610      1620      1630      1640      1650
CAMKKMmu,/1-251   ---------------------------------------------------------------------
NrkCfa,/1-258     ---------------------------------------------------------------------
Nrk6Cre,/1-257    ---------------------------------------------------------------------
Nek7simCel,/1-266 ---------------------------------------------------------------------
NEK6Hs,/1-266     ---------------------------------------------------------------------
NEK7Hs,/1-265     ---------------------------------------------------------------------
Nek82Cel,/1-264   ---------------------------------------------------------------------
NEK9Nercc1,/1-257 ---------------------------------------------------------------------
NRK16Tth,/1-256   ---------------------------------------------------------------------
NRK24Tth,/1-256   ---------------------------------------------------------------------
Nek81Cel,/1-254   ---------------------------------------------------------------------
Nek8simDme,/1-254 ---------------------------------------------------------------------
Fin1Spo,/1-278    ---------------------------------------------------------------------
TvSTEPK/1-307     ---------------------------------------------------------------------
TvAGCPK/1-307     ---------------------------------------------------------------------
KIN3Sce,/1-323    ---------------------------------------------------------------------
Nek2simDme,/1-263 ---------------------------------------------------------------------
Ng29678/1-363     ---------------------------------------------------------------------
NIM1Ncr,/1-284    ---------------------------------------------------------------------
NIMAAnid,/1-285   ---------------------------------------------------------------------
NRK5Tth,/1-259    ---------------------------------------------------------------------
NRK4Tth,/1-257    ---------------------------------------------------------------------
NRK2Tth,/1-257    ---------------------------------------------------------------------
NRK31Ttha,/1-263  ---------------------------------------------------------------------
NRK1Tth,/1-259    ---------------------------------------------------------------------
NRK3Tth,/1-258    ---------------------------------------------------------------------
NRK7Tth,/1-257    ---------------------------------------------------------------------
NRK9Tth,/1-273    ---------------------------------------------------------------------
13981lc/1-1109    ----VEAARILVNHEANMRD-----------------KYDETALMKAAKSGAVNVARILLPVEACQTRY
17231lc/1-1006    ----PDIANTLLKDEACMRD-----------------LHEETAMMKAAALGAYDVVKVLIDSEGGSRRP
24321disc/1-288   ---------------------------------------------------------------------
17250/1-854       ----IDNRTCLMIAALSRSLSCVRVLAEQRIGMGCQDGKGSTALIMAALSGYADICKVLAPLEARIQRK
15409/1-515       ---------------------------------------------------------------------
11775/1-1618      SLMEAALAGSLDAAKILVDHEHGMRARARFVVGNSYFDEATALMFAAAAGHESIVRLLIPYECKMHESC
8445/1-597        ---------------------------------------------------------------------
11311_mp/1-367    ---------------------------------------------------------------------
95593/1-376       ---------------------------------------------------------------------
```

```
                          1660      1670      1680      1690      1700      1710      1720
CAMKKMmu,/1-251     ---------------------------------------------------------------------
NrkCfa,/1-258       ---------------------------------------------------------------------
Nrk6Cre,/1-257      ---------------------------------------------------------------------
Nek7simCel,/1-266   ---------------------------------------------------------------------
NEK6Hs,/1-266       ---------------------------------------------------------------------
NEK7Hs,/1-265       ---------------------------------------------------------------------
Nek82Cel,/1-264     ---------------------------------------------------------------------
NEK9Nercc1,/1-257   ---------------------------------------------------------------------
NRK16Tth,/1-256     ---------------------------------------------------------------------
NRK24Tth,/1-256     ---------------------------------------------------------------------
Nek81Cel,/1-254     ---------------------------------------------------------------------
Nek8simDme,/1-254   ---------------------------------------------------------------------
Fin1Spo,/1-278      ---------------------------------------------------------------------
TvSTEPK/1-307       ---------------------------------------------------------------------
TvAGCPK/1-307       ---------------------------------------------------------------------
KIN3Sce,/1-323      ---------------------------------------------------------------------
Nek2simDme,/1-263   ---------------------------------------------------------------------
Ng29678/1-363       ---------------------------------------------------------------------
NIM1Ncr,/1-284      ---------------------------------------------------------------------
NIMAAnid,/1-285     ---------------------------------------------------------------------
NRK5Tth,/1-259      ---------------------------------------------------------------------
NRK4Tth,/1-257      ---------------------------------------------------------------------
NRK2Tth,/1-257      ---------------------------------------------------------------------
NRK31Ttha,/1-263    ---------------------------------------------------------------------
NRK1Tth,/1-259      ---------------------------------------------------------------------
NRK3Tth,/1-258      ---------------------------------------------------------------------
NRK7Tth,/1-257      ---------------------------------------------------------------------
NRK9Tth,/1-273      ---------------------------------------------------------------------
13981lc/1-1109      DGKTALMCAAEAGHLPLVSLLLPKEGRMKRTDGTTALMCAAQSNCIEAVRLLLPSEKRMQKLDGETALI
17231lc/1-1006      DGKTALMCAADAGYADIVGSLMNRESRMRKNDGTTALMYATERGHTECVRLLVETEKCIQRQNGETALM
24321disc/1-288     ---------------------------------------------------------------------
17250/1-854         DGFTALMSAAQEGHKECVSILLSKEGDVHKPGGWTALMSAALKNKPEIVQILSKDKRQIRQKREKDGIT
15409/1-515         ---------------------------------------------------------------------
11775/1-1618        NGKTALMAAAAAGHLSTLKLLIDSEGGLTGQNGWTALMSVVSKQYVDCVAPLLEKEAGMRDSQGNCALV
8445/1-597          ---------------------------------------------------------------------
11311_mp/1-367      ---------------------------------------------------------------------
95593/1-376         ---------------------------------------------------------------------
```

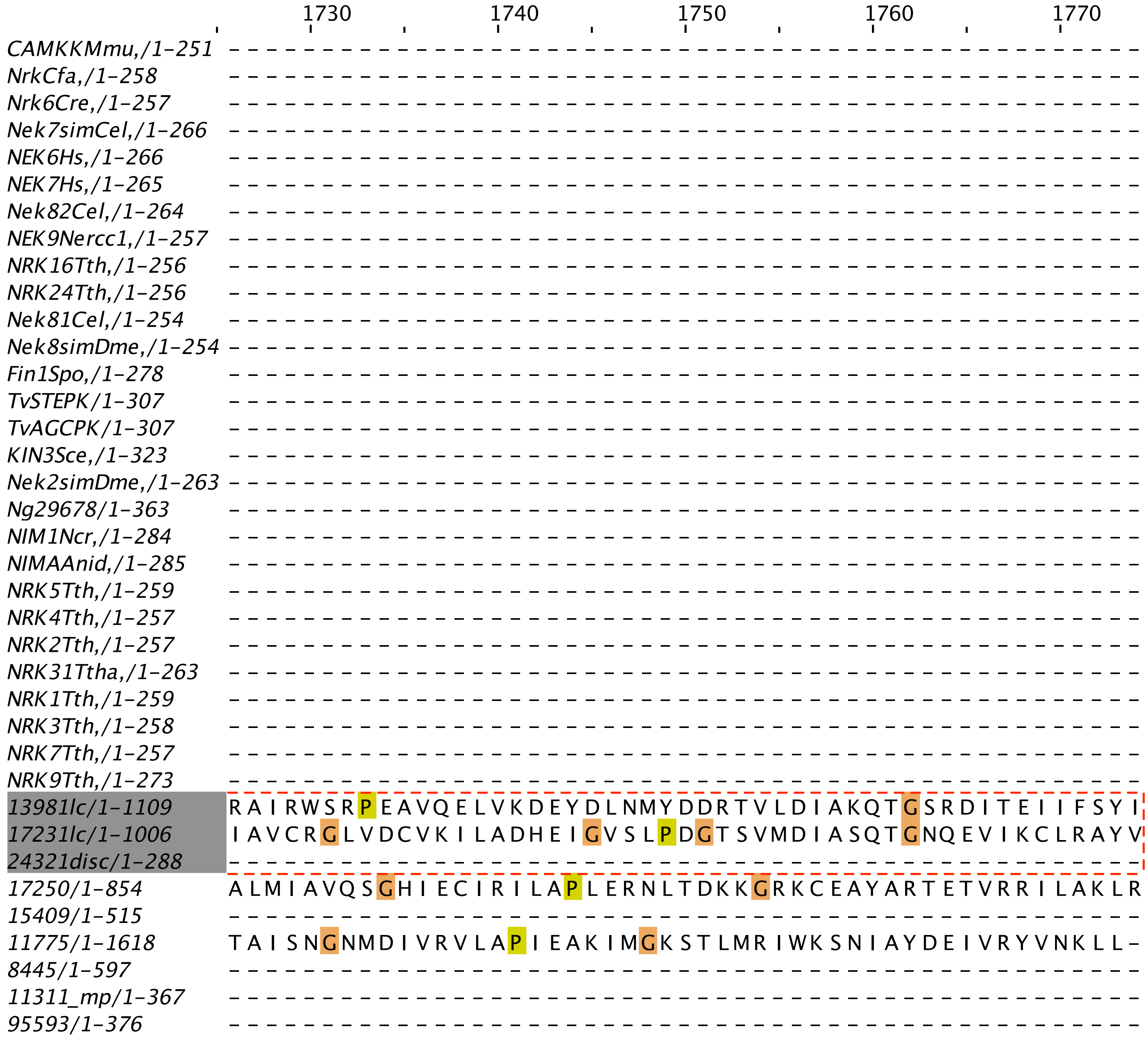

1730
1740
1750
1760
1770
CAMKKMmu,/1-251
NrkCfa,/1-258
Nrk6Cre,/1-257
Nek7simCel,/1-266
NEK6Hs,/1-266
NEK7Hs,/1-265
Nek82Cel,/1-264
NEK9Nercc1,/1-257
NRK16Tth,/1-256
NRK24Tth,/1-256
Nek81Cel,/1-254
Nek8simDme,/1-254
Fin1Spo,/1-278
TvSTEPK/1-307
TvAGCPK/1-307
KIN3Sce,/1-323
Nek2simDme,/1-263
Ng29678/1-363
NIM1Ncr,/1-284
NIMAAnid,/1-285
NRK5Tth,/1-259
NRK4Tth,/1-257
NRK2Tth,/1-257
NRK31Ttha,/1-263
NRK1Tth,/1-259
NRK3Tth,/1-258
NRK7Tth,/1-257
NRK9Tth,/1-273
13981lc/1-1109 RAIRWSRPEAVQELVKDEYDLNMYDDRTVLDIAKQTGSRDITEIIFSYI
17231lc/1-1006 IAVCRGLVDCVKILADHEIGVSLPDGTSVMDIASQTGNQEVIKCLRAYV
24321disc/1-288
17250/1-854 ALMIAVQSGHIECIRILAPLERNLTDKKGRKCEAYARTETVRRILAKLR
15409/1-515
11775/1-1618 TAISNGNMDIVRVLAPIEAKIMGKSTLMRIWKSNIAYDEIVRYVNKLL-
8445/1-597
11311_mp/1-367
95593/1-376
