# Supplementary material for: Novel Structural Components of the Ventral Disc and Lateral Crest in Giardia intestinalis
Source: PLoS Negl Trop Dis. 2011 Dec 20;5(12):e1442. doi: 10.1371/journal.pntd.0001442 (PMC3243723; doi:10.1371/journal.pntd.0001442)
Supplement: Figure S2 — Multiple sequence alignment of DIP13 homologs. The Giardia DIP13 homolog was aligned to DIP13 homologs in other representative eukaryotes using MUSCLE [75]. Alignment is presented using JalView [76]. (PDF) [file pntd.0001442.s002.pdf]

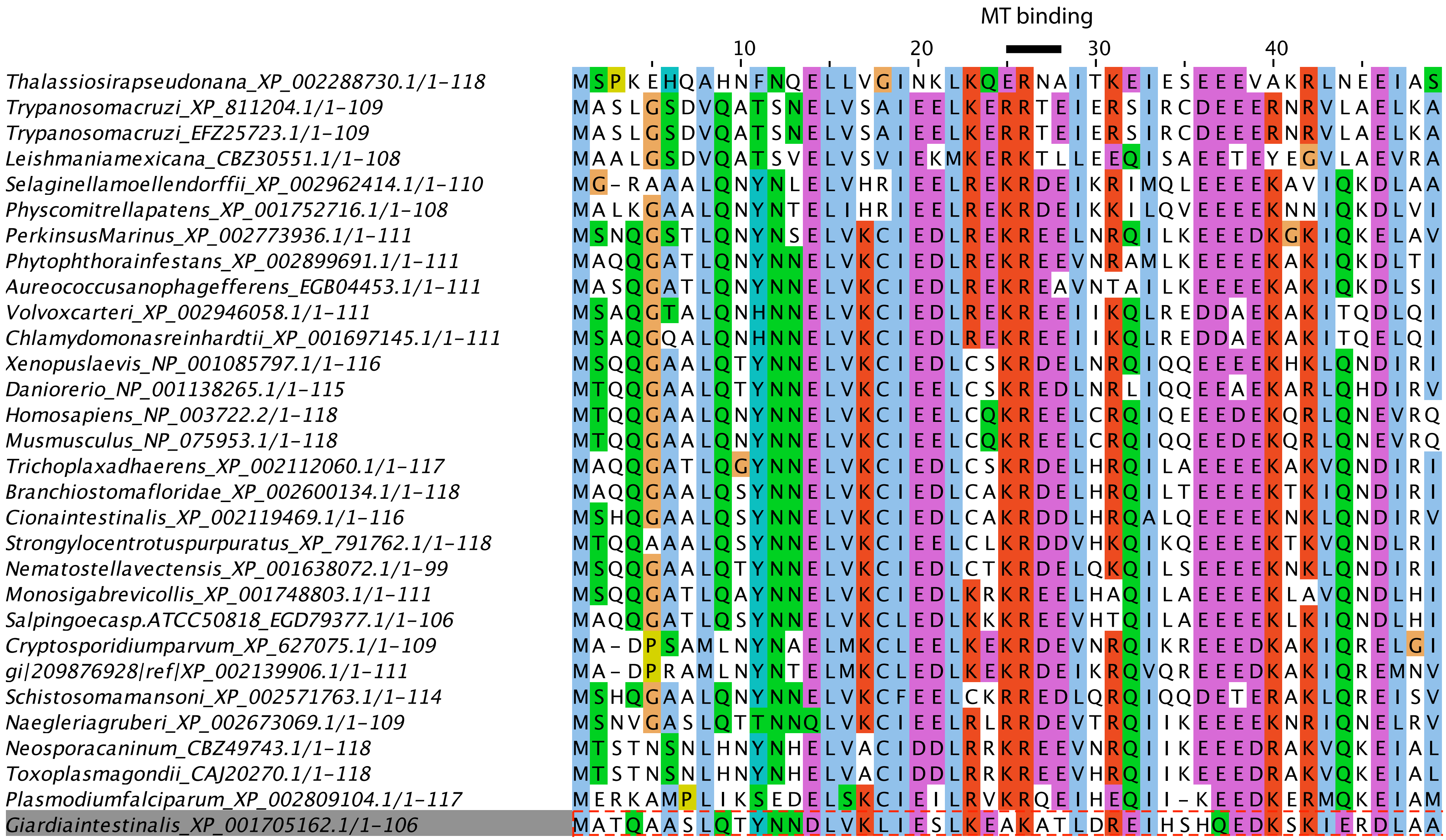

MT binding
10
20
30
40
Thalassiosirapseudonana_XP_002288730.1/1-118 MSPKEHQAHNFNQELLVGINKLKQERNAITKEIESEEEVAKRLNEEIAS
Trypanosomacruzi_XP_811204.1/1-109 MASLGSDVQATSNELVSAIEELKERRTEIERSIRCDEEERNRVLAELKA
Trypanosomacruzi_EFZ25723.1/1-109 MASLGSDVQATSNELVSAIEELKERRTEIERSIRCDEEERNRVLAELKA
Leishmaniamexicana_CBZ30551.1/1-108 MAALGSDVQATSVELVSVIEKMKERKTLLEEQISAEETEYEGVLAEVRA
Selaginellamoellendorffii_XP_002962414.1/1-110 MG-RAAALQNYNLELVHRIEELREKRDEIKRIMQLEEEEKAVIQKDLAA
Physcomitrellapatens_XP_001752716.1/1-108 MALKGAALQNYNTELIHRIEELREKRDEIKKILQVEEEEKNNIQKDLVI
PerkinsusMarinus_XP_002773936.1/1-111 MSNQGSTLQNYNSELVKCIEDLREKREELNRQILKEEEDKGKIQKELAV
Phytophthorainfestans_XP_002899691.1/1-111 MAQQGATLQNYNNELVKCIEDLREKREEVNRAMLKEEEEKAKIQKDLTI
Aureococcusanophagefferens_EGB04453.1/1-111 MASQGATLQNYNNELVKCIEDLREKREAVNTAILKEEEEKAKIQKDLSI
Volvoxcarteri_XP_002946058.1/1-111 MSAQGTALQNHNNELVKCIEDLREKREEIIKQLREDDAEKAKITQDLQI
Chlamydomonasreinhardtii_XP_001697145.1/1-111 MSAQGQALQNHNNELVKCIEDLREKREEIIKQLREDDAEKAKITQELQI
Xenopuslaevis_NP_001085797.1/1-116 MSQQGAALQTYNNELVKCIEDLCSKRDELNRQIQQEEEEKHKLQNDIRI
Daniorerio_NP_001138265.1/1-115 MTQQGAALQTYNNELVKCIEELCSKREDLNRLIQQEEAEKARLQHDIRV
Homosapiens_NP_003722.2/1-118 MTQQGAALQNYNNELVKCIEELCQKREELCRQIQEEEDEKQRLQNEVRQ
Musmusculus_NP_075953.1/1-118 MTQQGAALQNYNNELVKCIEELCQKREELCRQIQQEEDEKQRLQNEVRQ
Trichoplaxadhaerens_XP_002112060.1/1-117 MAQQGATLQGYNNELVKCIEDLCSKRDELHRQILAEEEEKAKVQNDIRI
Branchiostomafloridae_XP_002600134.1/1-118 MAQQGAALQSYNNELVKCIEDLCAKRDELHRQILTEEEEKTKIQNDIRI
Cionaintestinalis_XP_002119469.1/1-116 MSHQGAALQSYNNELVKCIEDLCAKRDDLHRQALQEEEEKNKLQNDIRV
Strongylocentrotuspurpuratus_XP_791762.1/1-118 MTQQAAALQSYNNELVKCIEELCLKRDDVHKQIKQEEEEKTKVQNDLRI
Nematostellavectensis_XP_001638072.1/1-99 MSQQGAALQTYNNELVKCIEDLCTKRDELQKQILSEEEEKNKLQNDIRI
Monosigabrevicollis_XP_001748803.1/1-111 MSQQGATLQAYNNELVKCIEDLKRKREELHAQILAEEEEKLAVQNDLHI
Salpingoecasp.ATCC50818_EGD79377.1/1-106 MAQQGATLQSYNNELVKCLEDLKKKREEVHTQILAEEEEKLKIQNDLHI
Cryptosporidiumparvum_XP_627075.1/1-109 MA-DPSAMLNYNAELMKCLEELKEKRDEVNRQIKREEEDKAKIQRELGI
gi|209876928|ref|XP_002139906.1/1-111 MA-DPRAMLNYNTELMKCLEDLKEKRDEIKRQVQREEEDKAKIQREMNV
Schistosomamansoni_XP_002571763.1/1-114 MSHQGAALQNYNNELVKCFEELCKRREDLQRQIQQDETERAKLQREISV
Naegleriagruberi_XP_002673069.1/1-109 MSNVGASLQTTNNQLVKCIEELRLRRDEVTRQIIKEEEEKNRIQNELRV
Neosporacaninum_CBZ49743.1/1-118 MTSTNSNLHNYNHELVACIDDLRRKREEVNRQIIKEEEDRAKVQKEIAL
Toxoplasmagondii_CAJ20270.1/1-118 MTSTNSNLHNYNHELVACIDDLRRKREEVHRQIIKEEEDRAKVQKEIAL
Plasmodiumfalciparum_XP_002809104.1/1-117 MERKAMPLIKSEDELSKCIEILRVKRQEIHEQII-KEEDKERMQKEIAM
Giardiaintestinalis_XP_001705162.1/1-106 MATQAASLQTYNNDLVKLIESLKEAKATLDREIHSHQEDKSKIERDLAA

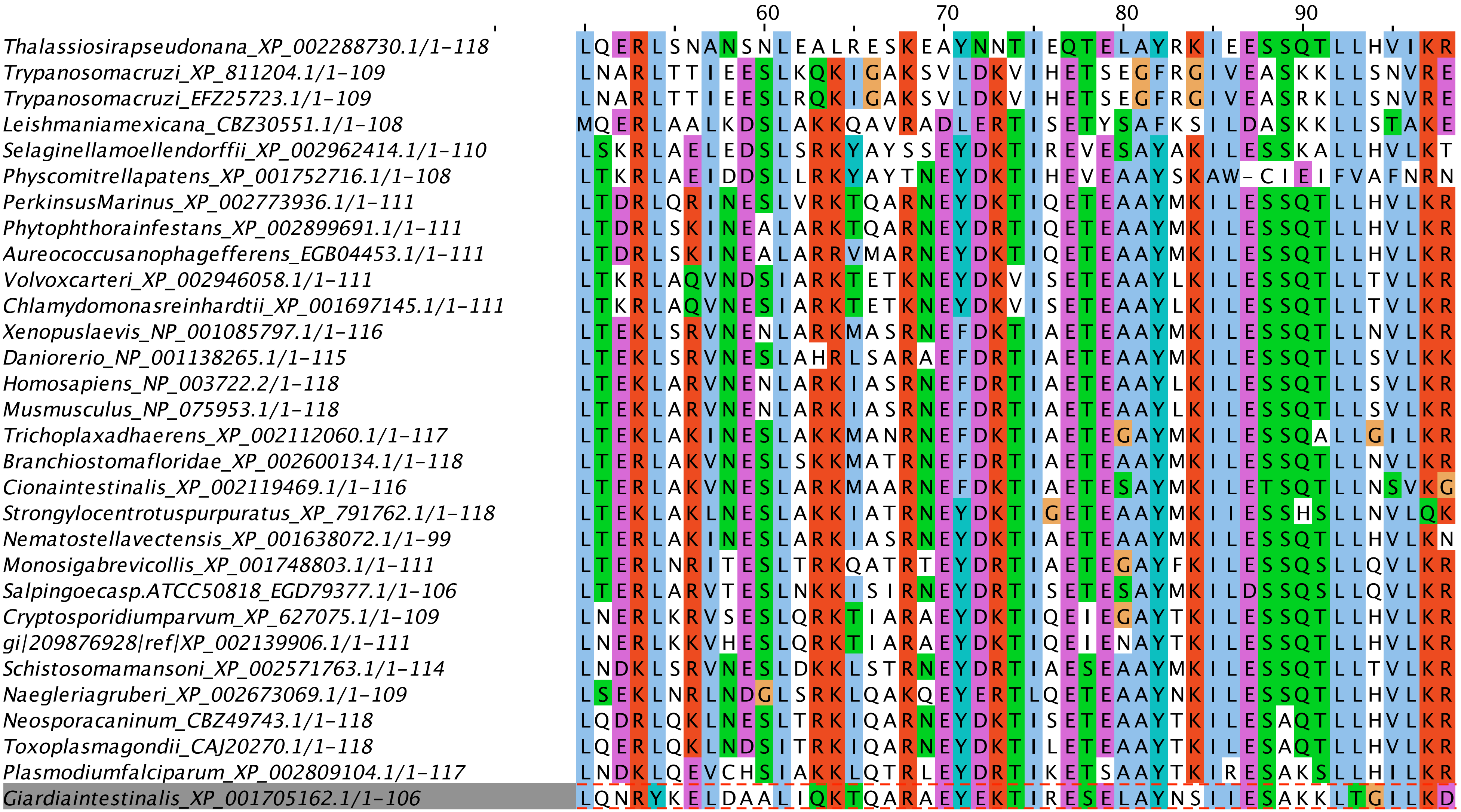
60 70 80 90
Thalassiosirapseudonana_XP_002288730.1/1-118 LQERLSNANSNLEALRESKEAYNNTIEQTELAYRKIEESSQTLLHVIKR
Trypanosomacruzi_XP_811204.1/1-109 LNARLTTIEESLKQKIGAKSVLDKVIHETSEGFRGIVEASKKLLSNVRE
Trypanosomacruzi_EFZ25723.1/1-109 LNARLTTIEESLRQKIGAKSVLDKVIHETSEGFRGIVEASRKLLSNVRE
Leishmaniamexicana_CBZ30551.1/1-108 MQERLAALKDSLAKKQAVRADLERTISETYSAFKSILDASKKLLSTAKE
Selaginellamoellendorffii_XP_002962414.1/1-110 LSKRLAELEDSLSRKYAYSSEYDKTIREVESAYAKILESSKALLHVLKT
Physcomitrellapatens_XP_001752716.1/1-108 LTKRLAEIDDSLLRKYAYTNEYDKTIHEVEAAYSKAW-CIEIFVAFNRN
PerkinsusMarinus_XP_002773936.1/1-111 LTDRLQRINESLVRKTQARNEYDKTIQETEAAYMKILESSQTLLHVLKR
Phytophthorainfestans_XP_002899691.1/1-111 LTDRLSKINEALARKTQARNEYDRTIQETEAAYMKILESSQTLLHVLKR
Aureococcusanophagefferens_EGB04453.1/1-111 LTDRLSKINEALARRVMARNEYDKTIQETEAAYMKILESSQTLLHVLKR
Volvoxcarteri_XP_002946058.1/1-111 LTKRLAQVNDSIARKTETKNEYDKVISETEAAYLKILESSQTLLTVLKR
Chlamydomonasreinhardtii_XP_001697145.1/1-111 LTKRLAQVNESIARKTETKNEYDKVISETEAAYLKILESSQTLLTVLKR
Xenopuslaevis_NP_001085797.1/1-116 LTEKLSRVNENLARKMASRNEFDKTIAETEAAYMKILESSQTLLNVLKR
Daniorerio_NP_001138265.1/1-115 LTEKLSRVNESLAHRLSARAEFDRTIAETEAAYMKILESSQTLLSVLKK
Homosapiens_NP_003722.2/1-118 LTEKLARVNENLARKIASRNEFDRTIAETEAAYLKILESSQTLLSVLKR
Musmusculus_NP_075953.1/1-118 LTEKLARVNENLARKIASRNEFDRTIAETEAAYLKILESSQTLLSVLKR
Trichoplaxadhaerens_XP_002112060.1/1-117 LTEKLAKINESLAKKMANRNEFDKTIAETEGAYMKILESSQALLGILKR
Branchiostomafloridae_XP_002600134.1/1-118 LTERLAKVNESLSKKMATRNEFDRTIAETEAAYMKILESSQTLLNVLKR
Cionaintestinalis_XP_002119469.1/1-116 LTERLAKVNESLARKMAARNEFDKTIAETESAYMKILETSQTLLNSVKG
Strongylocentrotuspurpuratus_XP_791762.1/1-118 LTEKLAKLNESLAKKIATRNEYDKTIGETEAAYMKIIESSHSLLNVLQK
Nematostellavectensis_XP_001638072.1/1-99 LTERLAKINESLARKIASRNEYDKTIAETEAAYMKILESSQTLLHVLKN
Monosigabrevicollis_XP_001748803.1/1-111 LTERLNRITESLTRKQATRTEYDRTIAETEGAYFKILESSQSLLQVLKR
Salpingoecasp.ATCC50818_EGD79377.1/1-106 LTERLARVTESLNKKISIRNEYDRTISETESAYMKILDSSQSLLQVLKR
Cryptosporidiumparvum_XP_627075.1/1-109 LNERLKRVSESLQRKTIARAEYDKTIQEIEGAYTKILESSQTLLHVLKR
gi|209876928|ref|XP_002139906.1/1-111 LNERLKKVHESLQRKTIARAEYDKTIQEIENAYTKILESSQTLLHVLKR
Schistosomamansoni_XP_002571763.1/1-114 LNDKLSRVNESLDKKLSTRNEYDRTIAESEAAYMKILESSQTLLTVLKR
Naegleriagruberi_XP_002673069.1/1-109 LSEKLNRLNDGLSRKLQAKQEYERTLQETEAAYNKILESSQTLLHVLKR
Neosporacaninum_CBZ49743.1/1-118 LQDRLQKLNESLTRKIQARNEYDKTISETEAAYTKILESAQTLLHVLKR
Toxoplasmagondii_CAJ20270.1/1-118 LQERLQKLNDSITRKIQARNEYDKTILETEAAYTKILESAQTLLHVLKR
Plasmodiumfalciparum_XP_002809104.1/1-117 LNDKLQEVCHSIAKKLQTRLEYDRTIKETSAAYTKIRESAKSLLHILKR
Giardiaintestinalis_XP_001705162.1/1-106 LQNRYKELDAALIQKTQARAEYEKTIRESELAYNSIIESAKKLTGILKD

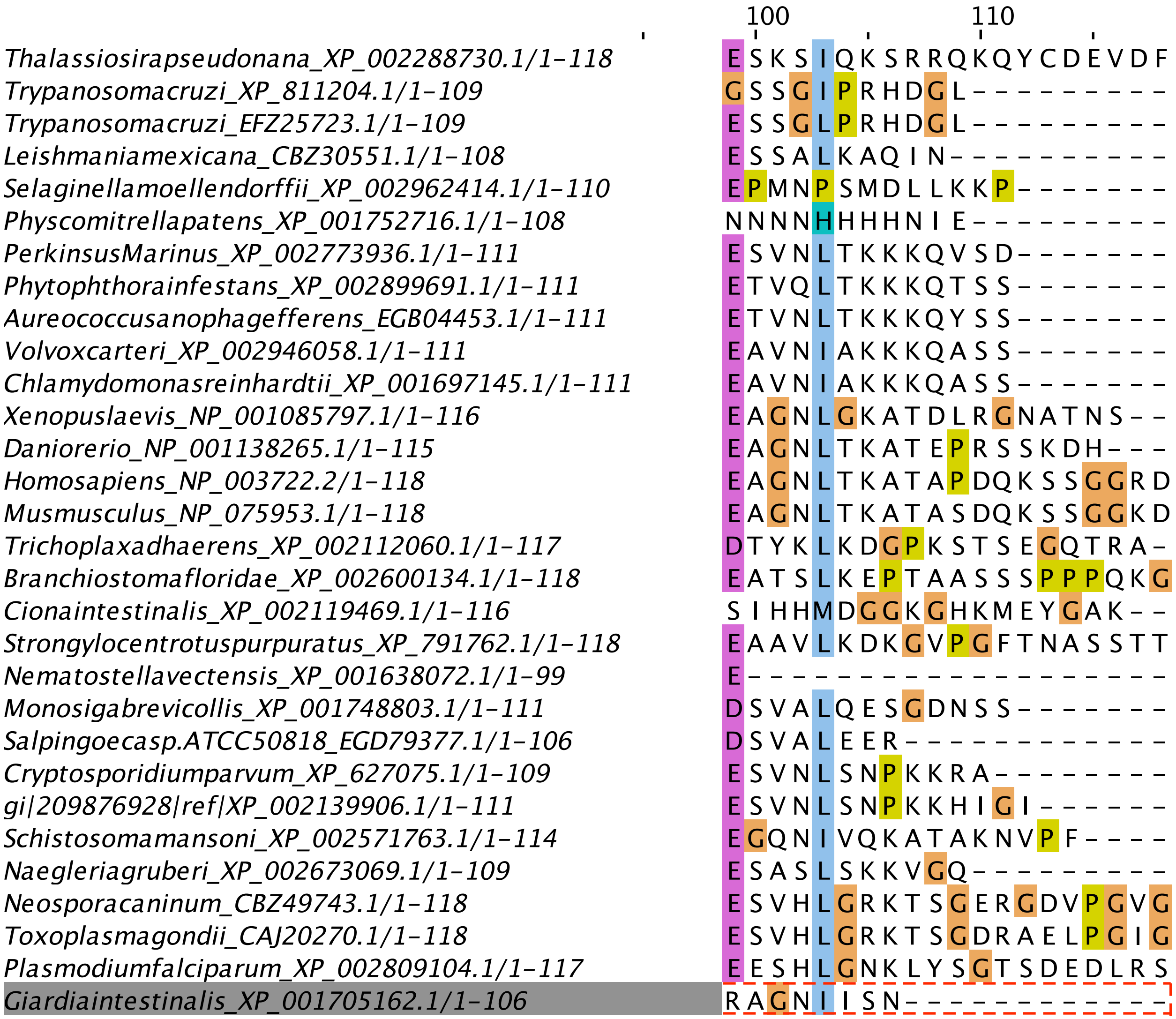
100
110
Thalassiosirapseudonana_XP_002288730.1/1-118 ESKSIQKSRRQKQYCDEVDF
Trypanosomacruzi_XP_811204.1/1-109 GSSGIPRHDGL---------
Trypanosomacruzi_EFZ25723.1/1-109 ESSGLPRHDGL---------
Leishmaniamexicana_CBZ30551.1/1-108 ESSALKAQIN----------
Selaginellamoellendorffii_XP_002962414.1/1-110 EPMNPSMDLLKKP-------
Physcomitrellapatens_XP_001752716.1/1-108 NNNNHHHHNIE---------
PerkinsusMarinus_XP_002773936.1/1-111 ESVNLTKKKQVSD-------
Phytophthorainfestans_XP_002899691.1/1-111 ETVQLTKKKQTSS-------
Aureococcusanophagefferens_EGB04453.1/1-111 ETVNLTKKKQYSS-------
Volvoxcarteri_XP_002946058.1/1-111 EAVNIAKKKQASS-------
Chlamydomonasreinhardtii_XP_001697145.1/1-111 EAVNIAKKKQASS-------
Xenopuslaevis_NP_001085797.1/1-116 EAGNLGKATDLRGNATNS--
Daniorerio_NP_001138265.1/1-115 EAGNLTKATEPRSSKDH---
Homosapiens_NP_003722.2/1-118 EAGNLTKATAPDQKSSGGRD
Musmusculus_NP_075953.1/1-118 EAGNLTKATASDQKSSGGKD
Trichoplaxadhaerens_XP_002112060.1/1-117 DTYKLKDGPKSTSEGQTRA-
Branchiostomafloridae_XP_002600134.1/1-118 EATSLKEPTAASSSPPPQKG
Cionaintestinalis_XP_002119469.1/1-116 SIHHMDGGKGHKMEYGAK--
Strongylocentrotuspurpuratus_XP_791762.1/1-118 EAAVLKDKGVPGFTNASSTT
Nematostellavectensis_XP_001638072.1/1-99 E-------------------
Monosigabrevicollis_XP_001748803.1/1-111 DSVALQESGDNSS-------
Salpingoecasp.ATCC50818_EGD79377.1/1-106 DSVALEER------------
Cryptosporidiumparvum_XP_627075.1/1-109 ESVNLSNPKKRA--------
gi|209876928|ref|XP_002139906.1/1-111 ESVNLSNPKKHIGI------
Schistosomamansoni_XP_002571763.1/1-114 EGQNIVQKATAKNVPF----
Naegleriagruberi_XP_002673069.1/1-109 ESASLSKKVGQ---------
Neosporacaninum_CBZ49743.1/1-118 ESVHLGRKTSGERGDVPGVG
Toxoplasmagondii_CAJ20270.1/1-118 ESVHLGRKTSGDRAELPGIG
Plasmodiumfalciparum_XP_002809104.1/1-117 EESHLGNKLYSGTSDEDLRS
Giardiaintestinalis_XP_001705162.1/1-106 RAGNIISN------------
