# Supplementary figures and images for: Novel Structural Components of the Ventral Disc and Lateral Crest in Giardia intestinalis
Source: PLoS Negl Trop Dis. 2011 Dec 20;5(12):e1442. doi: 10.1371/journal.pntd.0001442 (PMC3243723; doi:10.1371/journal.pntd.0001442)

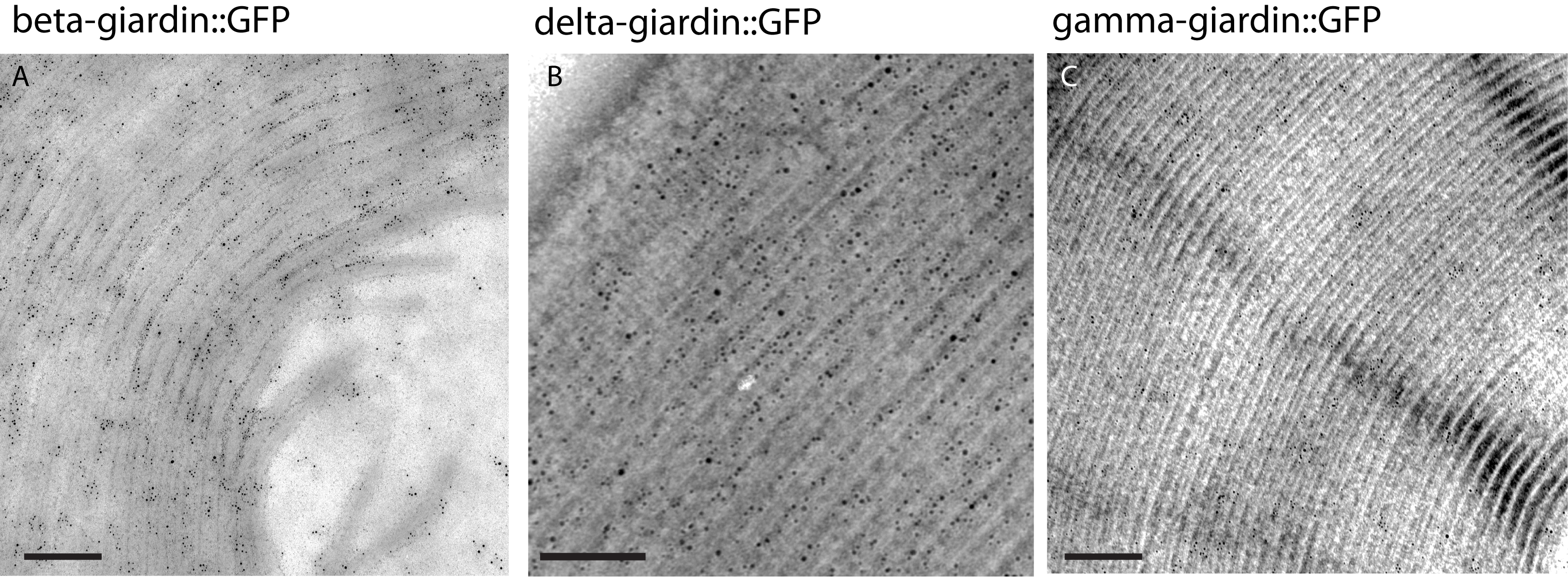

Supplement: Figure S3 — Beta-, gamma-, and delta-giardin localize to the microribbons. Negative staining using anti-GFP immunogold labeling of beta-giardin::GFP, delta-giardin::GFP and gamma-giardin::GFP strains show the association of these proteins with the ventral disc microribbons. Scale bar = 200 nm. (TIF) [file pntd.0001442.s003.tif]
