# Supplementary material for: Novel Structural Components of the Ventral Disc and Lateral Crest in Giardia intestinalis
Source: PLoS Negl Trop Dis. 2011 Dec 20;5(12):e1442. doi: 10.1371/journal.pntd.0001442 (PMC3243723; doi:10.1371/journal.pntd.0001442)
Supplement: Table S1 — Gateway and other oligonucleotide PCR primers for disc-associated proteins (DAPs). List of forward and reverse oligonucleotide PCR primers used in the construction of the DAP::GFP Giardia strains. (PDF) [file pntd.0001442.s004.pdf]

GATEWAY and other primers for disc-associated proteins (DAPs)

GiardiaDB	Forward primer sequence (5' to 3')	Reverse primer sequence (5 to 3')
GL50803_4410	CACCAGGAAAGCGTTGGTGTCAAGAACC	CAGGACACCCACTCCCGTG
GL50803_4812*	GATCGGCGCGCCATGTCTATGTTCACCTCCACCCGTACGCTCAC	ACCGGTAGAGCGCCGGCTCGGGCTCCGGCCGCTGCGCCGTGCTTTGTGACCATCGAGAG
GL50803_5374	CACCGCTTAGCACACACAAAACAGTATATTACAT	CAGGTCCGAAATGGAGCTGTCTAG
GL50803_6417	CACCACCACAACCTGCATACGTGTGG	AGTCTGCACTATGTTATTGTACAGTCCGT
GL50803_7203	CACCGGGACCGTCACTGTCATTCATCAG	CCCCGAGAGGAATCTCCGCA
GL50803_8445	CACCTGGGTTGCAAACGCCCTG	CTTGTTGTTAGTCTGTAGCCATGTTCTTAT
GL50803_9030	CACCGCAATTTTCGAAGCTGTATGGGTCT	AACATTCTTAGAGTCGGCCATGGACAAAATCA
GL50803_9909	CACCAGATAAGCAACCCTTCGAGTGCC	CTGGTTAGTGCGAGCCTTGATAGC
GL50803_11311	CACCAGCCAAATGCAATTCTAATCAATTAAAGCT	ATAGCCTTTATATGTTCCGGCGTTCAG
GL50803_11390	CACCTGGGTACATCGACAAGTACACTACCG	ACAGGCGGATGAACCACTGAG
GL50803_13766	CACCGTCCCAGCTGTTTAGCTTTTCGACA	AAAGCCTAGCCCTCCGTACTG
GL50803_13981	CACCATTTAGAGCGTGGGGAAAGTACATAGC	TATATAAGAGAATATTATCTCTGTAATGTC
GL50803_14270	CACCGACAAGTCATAGTAAATGCAGCAGATATGG	ATCCGTCTGGGGCTTTTGTATGAC
GL50803_14872	CACCTCAGTAGCTTAGGATGGGTTCTCCC	AATAGGCCTCCACGGGGC
GL50803_15054	CACCGAGACGCGTGGCCAAAGAGC	CTGCATGAACTCCAGCGCAAG
GL60803_15101	CACCAGCAATCTCTTATTTACTCAACCAGTTTAT	TGCCTCGGGAGCCCAAAG
GL50803_15409	CACCCTCCCTACGCACACGTCCCGGAAGAT	CAGGTTCAGGACCTCACGCACTCTGCTGCTC
GL50803_15427	CACCCAGAACAAACTGTTCAAGAGTCTGGTCA	AAAGCCTATCTTGATCTGCCTAATGAACC
GL50803_15456	CACCACGCTCCGTTGGTTGTTAGTGC	ATAGGCTTTGGTTAGTATCTCTTTAATACG
GL50803_15576	CACCGAATTGGATAGTAAGCTAGAGGCGCTAAAC	TTTTCTGGATGCCTTCTTTCCTTTAGTAAG
GL50803_15993	CACCCTCTAAAAAGAGCAGGAGACCTATAAAACA	TAAATATTTCCCCATCATCTCCTGCAACAT
GL50803_15995	CACCCTCCGGAGCCTCTCCTGAGC	CTTGGTTCGTAAGCGGCCTCC
GL50803_16220	CACCTATTAATGCTAGCCCTTCGGACACAGA	GATGTGGTTTTCTTGGTTGCTTAGAAGC
GL50803_16263	CACCCCCATAGGCCCCCTTCCC	GTTGCTGATAATGTTACCCGCTCTATCC
GL50803_16343*	GGCGCGCCATGTCCGAGGCTATGGTTTTCAGCAAGATCG	ACCGGTAGCCCTGCCCCTGCCCCTGCCGCCGCGAACTTACCACTTCGGCCC
GL50803_16424	CACCCCCAGTAGTAAATACCCCAAGATTTGTCTG	GTAGCGACGATTACCGGATGAGTG
GL50803_16650*	AATTGGCGCGCCAGTGTTTGTCCCGTCTTACTCTGTTTTGTATAAG	ATCGAACCGGTAGGCTTTTCTGACGTGCTCCAAGCATCGGCAGCCTTG
GL50803_16745	CACCAGGGTATGAGACTGAGAAGTGGCT	TTGTGGCTGAATAGACGCTAGGTTG
GL50803_17053	CACCATACATCCATCGGTTAAAAGAACCTAATT	TATCAAGTGCTGCGCCTGTCTG
GL50803_17090	CACCCATCGTGTCGCCGCGTAGAAATC	CGCGAGCTCGACCTCGAC
GL50803_17096	CACCACGGAGAAATTCATAGCGCGGAC	AAGAACGCGGGGGTGTCTG
GL50803_17097	CACCTGCACACTGACAACATCGTGTCG	AGGACGGTAGTTGTTGATGCACTCGAGGATGGT
GL50803_17230*	ATAAGCCGCGCCATGAAGTCATCGTTCTCGACCGTCAGCTCC	ACCGGTAGAGCGCCGGCTCCGGCTCCGGCCGCTGCGCCATCAACCTTCGTCGTCATGATC
GL50803_17231	CACCCTGCACAACATACTTGTCGTTTCACATC	GACATACGCTCGAAGGCATTTGATCA
GL50803_17249	CACCAGCAGGACATCTACAAGAGGCTCA	ATCAAAGATTTCTAGAAGAGACGGGGC
GL50803_17250	CACCCAATGAAGGCATAGACCCCGAAAATTACT	CCCGAAGTTTAGCCAGGATCCTCCGCACGGTCT
GL50803_17536	CACCTATCTCCTCGCTGCTCTCCCTT	GCTCATTATGTTTCGCAAGGTCCG
GL50803_17585	CACCGCGTCCTCTCCCTGGCC	CCTTCTTCTTTCATACCCGGACATGTAATC
GL50803_17586	CACCTGGTGCGCCGTTATGAGCG	TATCCCAGTCATATCCAGAAGAAGACGG
GL50803_23492	CACCAACAGCTTCTGCACCATGGCT	CATCTTGCGAAGGATTGCCACCA
GL50803_24194	CACCGAGCGGCAGAGATGAAGCTTTTCT	CACTCGTGTTGTCTTGCTGGATACTC
GL50803_24321	CACCTCTATGTAGCTGTATGGACGCGGT	AGCTGAAAGCTCGCCCCTC
GL50803_24842	CACCGAGGAGAACCCCTGGAGTACTTGC	CGGAGAAAGAATGCTATCAGCCTCC
GL50803_86676*	ATAAGGCGCGCCATGGTCAGGGCCAAATGCGCGCCTTCTGC	ACCGGTAGAGCGCCGGCTCCGGCTCCGGCCGCTGCGCCGAAGACAAACTTCTCCTCCTGG
GL50803_87817	CACCGTTTGACCCCAGTGAGGAGGATG	CTGATAAGGTTCATGGCATAAAGGGCA
GL50803_91354	CACCGAGCTTTGCCCGCTTGTGAAG	ACGCCACCTTCGTTTTGTACGT
GL50803_101906	CACCCAGGTCAGCAGGGCCTGAG	GGTGGAGCCGAACTCCGC
GL50803_102455	CACCCCATTGGGTCCCTGGATG	CTCCATATGCGACGTTTCTTTGTTC
GL50803_103285	CACCAAAGGGGTACAGTAGCAGCGATG	CACCCCAGCACTGACCAGG
GL50803_103807	CACCATTTACTGAAACTTCTGTTTACTCGTTGAA	GTGGGTGCCAGACCCTTCTTC
GL50803_103810	CACCGTACTTAGTGGTTTTTTGCTCTGGCAATC	GACGGATCTGCTAGACAGAAGAGAGC
GL50803_112729*	ATTAGGCGCGCCGTGCAATGGAGCTGCTTGAAGAATGCACGCCAG	ATCGAACCGGTAGCTTATGGTATATTTCGATCCGTGACTGTCTTGT
GL50803_137716	CACCACGCCGGCCGCATCTG	CTGGAGGTCCAGGGCCG

*These primers were used to generate amplicons for cloning into pGFP.pac (Dawson, 2007)
